# A Tutorial on Bayesian Multi‐Study Factor Analysis With Applications in Nutrition and Genomics

**DOI:** 10.1002/sim.70531

**Published:** 2026-04-24

**Authors:** Mavis Liang, Blake Hansen, Alejandra Avalos‐Pacheco, Roberta De Vito

**Affiliations:** ^1^ Department of Biostatistics Brown University Providence RI USA; ^2^ Institute of Applied Statistics Johannes Kepler University Linz Linz Austria; ^3^ Harvard‐MIT Center for Regulatory Science Harvard Medical School Boston MA USA; ^4^ Dipartimento di Scienze Statistiche Sapienza University of Rome Rome Italy

**Keywords:** Bayesian statistics, factor analysis, genomics, integrative analysis, multi‐study, nutrition

## Abstract

High‐dimensional data are crucial in biomedical research. Integrating such data from multiple studies is a critical process that relies on the choice of advanced statistical models, enhancing statistical power, reproducibility, and scientific insight compared to analyzing each study separately. Factor analysis (FA) is a core dimensionality reduction technique that models observed data through a small set of latent factors. Bayesian extensions of FA have recently emerged as powerful tools for multi‐study integration, enabling researchers to disentangle shared biological signals from study‐specific variability. In this tutorial, we provide a practical and comparative guide to seven advanced Bayesian integrative factor models: Perturbed Factor Analysis (PFA), Bayesian Factor Regression with non‐local spike‐and‐slab priors (MOM‐SS), Subspace Factor Analysis (SUFA), Bayesian Multi‐study Factor Analysis (BMSFA), a variational‐inference implementation of BMSFA (CAVI), Bayesian Latent Analysis through Spectral Training (BLAST), and Bayesian Combinatorial Multi‐study Factor Analysis (Tetris). To contextualize these methods, we also include two benchmark approaches: Standard FA applied to pooled data (Stack FA), and FA applied separately to each study (Ind FA). We evaluate all methods through extensive simulations, assessing computational efficiency, accuracy in estimation of loadings, and the number of factors. To bridge theory and practice, we present a full analytical workflow—with detailed R code—demonstrating how to apply these models to real‐world datasets in nutrition and genomics. This tutorial is designed to guide applied researchers through the landscape of Bayesian integrative factor analysis, offering insights and tools for extracting interpretable, robust patterns from complex multi‐source data. Simulation code, R package “bmfaToolkits” and a user‐friendly guidebook can be found at https://github.com/Mavis‐Liang/Bayesian_integrative_FA_tutorial.

**Abbreviations**
BMSFA: Bayesian multi‐study factor analysis
CAVI: variational‐inference implementation of Bayesian multi‐study factor analysis
FA: factor analysis
MOM‐SS: Bayesian factor regression with non‐local spike‐and‐slab priors
PFA: perturbed factor analysis
SUFA: subspace factor analysis
Tetris: Bayesian combinatorial multi‐study factor analysis

## 1 Introduction

The rapid evolution of high‐throughput biological technologies has transformed biomedical research by producing large‐scale, complex, and heterogeneous data sets in different studies, platforms, and populations. Developing scalable and interpretable methods for their integrative analysis is critical to capture deep biological insights from such data, but their complexity demands advanced statistical frameworks that can go beyond standard approaches [1, 2]. Integration of such different studies is essential to improve statistical power, estimate reproducibility, and facilitate the discovery of robust biological signals by accounting for shared structures between datasets while modeling study‐specific variation [3, 4]. However, integrating data from diverse origins is far from trivial: Batch effects, platform‐specific biases, and population heterogeneity introduce challenges that require sophisticated statistical modeling [4].

Dimensionality reduction techniques, such as Principal Component Analysis (PCA) and Factor Analysis (FA), are foundational tools in high‐dimensional data analysis. These methods reduce complexity, facilitate visualization, and identify important latent structures underlying observed variables [5].

A broad class of *non‐Bayesian* (frequentist or optimization‐based) methods has also been developed for integrative analysis across studies or modalities, and it is useful to position our scope relative to this literature. For instance, Multiple Co‐Inertia Analysis (MCIA) [6] generalizes Co‐Inertia Analysis (CIA) [7] to more than two datasets by extracting study‐specific low‐dimensional representations and then maximizing their common structure. Multiple Factor Analysis (MFA) [8] extends PCA by combining study‐specific PCAs through a global compromise representation, and related ideas include meta‐analytic PCA approaches that identify a common linear subspace across studies [9]. Building on MSFA, Multi‐Study Factor Regression (MSFR) [10] Other integrative frameworks emphasize clustering or subtype discovery across multiple datasets [11, 12]. A particularly influential decomposition for multi‐block data is Joint and Individual Variation Explained (JIVE) [13], which separates joint and block‐specific structure and has motivated a substantial body of subsequent non‐Bayesian work (see, e.g., [14]).

While these non‐Bayesian approaches are often computationally efficient and widely used, they typically rely on point estimation with rank/penalty selection via heuristics, cross‐validation, or information criteria, and they may provide limited uncertainty quantification for latent structure and factor selection in complex, high‐dimensional settings. In contrast, Bayesian formulations offer a coherent mechanism to incorporate shrinkage/sparsity priors, borrow information across studies through hierarchical modeling, and quantify uncertainty in both factor dimensionality and loadings. For these reasons—and to keep the tutorial focused and practically actionable, we concentrate on Bayesian integrative factor‐analysis models for multi‐study high‐dimensional data, and we view the non‐Bayesian literature as complementary but outside the scope of this tutorial.

Also, some modern biological settings assume *count*‐valued data (e.g., mutations, RNA‐seq, and single‐cell assays). For such settings, alternative low‐rank decompositions have been developed. Examples include non‐negative matrix factorization(NMF) [15, 16, 17] and related Poisson/negative‐binomial factorization models, as well as other approaches tailored to count data integration and latent structure discovery [18, 19]. We restrict attention here to continuous high‐dimensional data and corresponding Gaussian factor models; extensions to count data are an important direction for future work.

In particular, Bayesian formulations of FA have proven powerful in high‐dimensional contexts: By leveraging priors that induce sparsity or shrinkage, such as spike‐and‐slab priors [20, 21, 22], cumulative shrinkage priors [23], or generalized sparse priors [24]: Bayesian FA enables more interpretable latent representations. These models also offer principled uncertainty quantification and automatic determination of the number of latent factors, making them attractive for complex biological applications [25].

Despite their utility, standard FA and PCA are not designed to handle the complexity of multi‐study data, where both shared and study‐specific sources of variation must be disentangled. Simple approaches like pooling data across studies (“stacking”) or analyzing each study separately (“independent analysis”) often obscure critical signals or amplify spurious artifacts, leading to misleading conclusions. In many modern applications—such as integrating gene expression data from different microarray platforms [26], harmonizing nutritional data from case‐control studies across populations [27], or modeling imaging data collected from multiple subjects and time points [28]—there is a clear need for statistical methods that can explicitly separate shared biological structure from study‐specific technical or biological variation.

To meet this need, multi‐study extensions of Bayesian FA have emerged. Multi‐Study Factor Analysis (MSFA) [29] and its Bayesian counterpart (BMSFA) [30, 31] jointly model common and study‐specific factors, using shrinkage priors to stabilize estimation in high dimensions. hlBayesian Latent Analysis through Spectral Training (BLAST) [32] builds on BMSFA by using spectral decompositions to separate shared and study‐specific factors, and estimates factor loadings and residual variances via surrogate Bayesian regression models. Perturbed Factor Analysis (PFA) [33] allows for structured study‐specific deviations via perturbation matrices. Bayesian Latent Factor Regression (e.g., MOM‐SS, Laplace‐SS) [34] combines spike‐and‐slab priors with batch adjustment mechanisms to isolate true biological signals. Subspace Factor Analysis (SUFA) [35] improves identifiability of shared and unique latent spaces, while Bayesian Combinatorial Multi‐Study FA (Tetris) [36] generalizes BMSFA by allowing flexible patterns of factor sharing across studies.

Although these methods offer remarkable flexibility and modeling power, they differ in terms of their assumptions, estimation strategies, and suitability for different types of high‐dimensional data. For applied researchers, it is often unclear which model to choose, how to interpret its output, or how to implement it correctly. Existing tutorials in the literature are often domain‐specific—for instance, focusing on genomic data and machine learning techniques [37], general data integration principles [38], or nutrition‐focused multi‐population studies [39]. However, none of these provide a comprehensive, statistically grounded tutorial focused on Bayesian integrative factor models for multi‐study high‐dimensional data. To fill this gap, we develop this tutorial to systematically evaluate and compare the most widely used Bayesian multi‐study factor models on a unified overview of these approaches. We provide theoretical and practical guidance on model assumptions, identifiability, computational considerations, and post‐processing. In doing so, we help researchers choose the most appropriate method based on their data characteristics and scientific objectives. Our tutorial is structured to support both conceptual understanding and direct application.

We focus on seven of the most prominent and cited Bayesian integrative factor models—PFA, MOM‐SS, SUFA, BMSFA, a variational‐inference implementation of BMSFA (CAVI), BLAST, and Tetris—alongside two benchmark approaches: Standard FA on pooled data (Stack FA) and standard FA applied separately to each study (Ind FA).

We compare these methods using extensive simulation studies that vary the number of studies, dimensionality, signal‐to‐noise ratio, and factor‐sharing structure. In addition, we demonstrate real‐world applications using genomic and nutritional datasets, with fully reproducible R code to guide implementation.

The remainder of the paper is organized as follows. In Section 2, we present the modeling framework for multi‐study factor analysis, detail each Bayesian method, and discuss identifiability and inference. Section 3 presents simulation studies comparing model performance across different settings. Section 4 illustrates the application of each method to real‐world datasets, with full analytical workflows and interpretation strategies. Finally, Section 5 concludes with practical recommendations and future directions.

## 2 Data Integration Methods

### 2.1 The Multi‐Study Data

**FIGURE 1:**
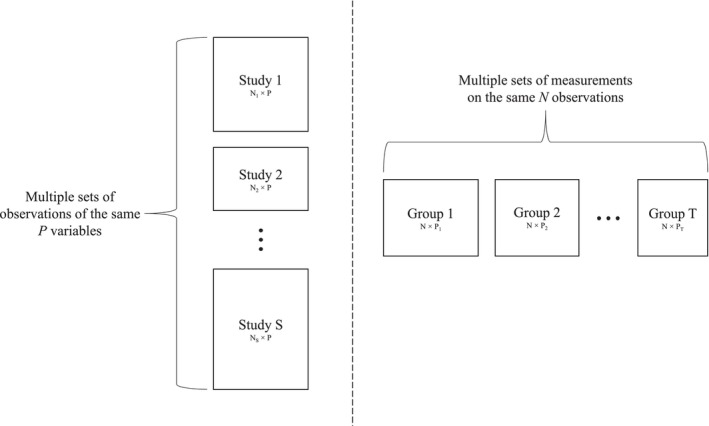
Data integration scenarios: Multi‐study data which observes the same set of variables across multiple groups of subjects (left), multi‐platform data which observes multiple sets of variables on the same subjects (right).

In this work, we focus on data integration techniques that provide insights into the data that single‐source analyses can miss. Specifically, there are two main model‐based data integration problems, as illustrated in Figure 1: (1) multi‐study data, where the same set of variables is measured across multiple independent studies or groups, and (2) multi‐platform (or multi‐modality) data, where different types of variables are collected on the same set of individuals.

When analyzing multi‐study data, the goal of analysis is two‐fold: To identify a common signal present within all of the studies, as well as to identify study‐specific signals unique to each study, or a combination of both. For example, in genomic studies, the aim is to identify robust, reproducible biological pathways that may be masked by batch effects in single‐study analyses. Integrating data across multiple cohorts can enhance statistical power and reveal robust shared components. Conversely, in some contexts such as nutritional epidemiology, the study‐specific effects—like dietary patterns specific to ethnic groups—are of primary interest.

The analysis of multi‐platform data aims to quantify different signals between distinct data sources and explore the intricacies of interconnections between those multiple layers. For instance, in oncology studies, multi‐modality data including electronic health records, molecular data, and medical images are often collected for the individuals [40]. While biomarker discoveries are mainly based on single modality molecular data, integrating different data modalities can improve disease classification and inform precision medicine strategies [40, 41]. Multi‐platform data present a different challenge, as we can no longer assume that the data are i.i.d. because the same set of subjects is observed under each platform. Consequently, statistical methods tailored for multi‐platform integration—such as Multi‐Omics Factor Analysis [42] or DIABLO [43]—have emerged to address these challenges.

In this tutorial, we focus on methods designed for the multi‐study setting, while recognizing that future extensions are needed to unify multi‐study and multi‐platform approaches under a common framework.

For simplicity, we use the term “multi‐study” to refer to datasets where the same set of P variables is measured across S independent groups, studies, or platforms. However, we emphasize that multi‐study can also be samples from different tissues or locations, replicated data collected in separate days or batches, or even experiments under different treatment conditions, which are subject to specific contexts.

To begin with, we introduce the general notation for the multi‐study setting used throughout this tutorial:

- S: Number of studies.
- $N_{s}$: Number of observations in the sth study, where s=1,…,S.
- P: Number of variables observed in each of the S studies.
- $y_{ips}$: Value for the pth variable of ith observation in sth study, for $i=1,\ldots ,N_{s}$, p=1,…,P, and s=1,…,S; with vector notation $y_{is}={\left(y_{i1},\ldots ,y_{iP}\right)}_{s}^{⊤}$.
- $Y_{s}={\left(y_{1s},\ldots ,y_{N_{s}s}\right)}^{⊤}$: The $N_{s}\times P$ data matrix for study s.

Figure 1 provides a visual comparison of the multi‐study and multi‐platform scenarios. In the remainder of this paper, we focus on integrative methods that assume each study provides measurements on the same variables, but across different samples. These settings are prevalent in genomics, nutrition, and other biomedical applications, and require careful statistical modeling to capture both common and study‐specific latent structures.

### 2.2 Simple Integrative Factor Approach

Factor analysis (FA) is a widely used technique for modeling high‐dimensional data $y_{i}$ through a smaller number of latent variables. We begin by introducing the standard FA model in the context of a single study, and then describe two naive strategies for applying this model to multi‐study data: Stack FA and Ind FA.

The classical factor model assumes: $y_{i}\in {\mathbb{R}}^{P}$ are modeled as:

(1) $$\begin{array}{c} \underset{\left(P\times 1\right)}{\underbrace{y_{i}}}=\underset{\left(P\times K\right)}{\underbrace{\Phi }}\;\underset{\left(K\times 1\right)}{\underbrace{f_{i}}}+\underset{\left(P\times 1\right)}{\underbrace{\epsilon_{i}}}, \end{array}$$

where Φ is the P×K factor loadings matrix, $f_{i}$ are a K‐dimensional independent *latent factor* vector, and $\epsilon_{i}$ is the *idiosyncratic residual* term. Typically, the model assumes:

(2) $$f_{i}∼\text{MVN}(0,I_{K}),\;\epsilon_{i}∼\text{MVN}(0,\Psi ),$$

where $\Psi =\text{diag}(\psi_{1},\ldots ,\psi_{P})$. Under independence between $f_{i}$ and $\epsilon_{i}$ and properties of the normal distribution, the marginal distribution of $y_{i}$ is:

(3) $$y_{i}∼\text{MVN}(0,\Phi \Phi^{⊤}+\Psi ).$$

Thus, the covariance structure is decomposed into a low‐rank loading component $\Phi \Phi^{⊤}$, capturing variability explained by the latent factors, and a diagonal matrix Ψ, capturing residual variances.

This model can also be expressed element‐wise as:

(4) $$y_{ip}=\overset{K}{\underset{k=1}{\sum }}\phi_{pk}f_{ik}+\epsilon_{ip},$$

where the loading $\phi_{pk}$ reflects the influence of factor k on variable p. Since the number of factors is less than the number of observed variables, K≪P, FA offers a compact and interpretable representation of high‐dimensional data.

Bayesian factor models focus on priors for factor loadings Φ, and residual covariance matrix Ψ. Flexible prior for the loadings include heavy‐tailed priors [44], spike‐and‐slab priors [20, 21, 22], and global‐local shrinkage priors such as the Multiplicative Gamma Process Shrinkage (MGPS) prior [23, 45]. These priors help control overfitting and enable automatic selection of the number of active factors.

Posterior inference proceeds via Gibbs sampling, Expectation‐Maximization, or variational methods. Selecting the number of latent factors K remains a challenging task, with methods ranging from information criteria [46] to fully Bayesian learning approaches [25, 47, 48]; see [49] for a comprehensive review.

#### 2.2.1 Stack FA

##### 2.2.1.1 Model

The first model for data integration relies on stacking all the individuals from the different studies into one combined matrix and fitting a single FA model (Stack FA), effectively ignoring study labels.

For each subject $i=1,\ldots ,N_{s}$ in study s=1,…,S, the model is:

(5) $$\underset{\left(P\times 1\right)}{\underbrace{y_{is}}}=\underset{\left(P\times K\right)}{\underbrace{\Phi }}\;\underset{\left(K\times 1\right)}{\underbrace{f_{is}}}+\underset{\left(P\times 1\right)}{\underbrace{\epsilon_{is}}},$$

where $f_{is}∼\text{MVN}(0,I_{K})$ are K‐dimensional latent factors, and $\epsilon_{is}∼\text{MVN}(0,\Psi )$ is the idiosyncratic error with $\Psi =\text{diag}(\psi_{1},\ldots ,\psi_{P})$. Because the same Φ and Ψ are shared across all studies, this model implies:

(6) $$\sum_{1}=\sum_{2}=\cdots =\sum_{S}=\Phi \Phi^{⊤}+\Psi .$$

Thus, Stack FA assumes that there is no systematic difference between studies. This homogeneous covariance assumption may be appropriate when the generative processes are similar across studies. However, it can mask meaningful heterogeneity if study‐specific structure exists.

##### 2.2.1.2 Priors

In this tutorial, we use the MGPS prior on Φ as proposed by Bhattacharya and Dunson [45]:

(7) $$\begin{array}{cc} \phi_{pk} & ∼𝒩(0,\omega_{pk}^{-1}\theta_{k}^{-1}),\\ \omega_{pk} & ∼\text{Gam}(\kappa /2,\kappa /2),\\ \theta_{k} & =\overset{k}{\underset{l=1}{\prod }}\delta_{l},\delta_{1}∼\text{Gam}(a_{1},1),\delta_{l}∼\text{Gam}(a_{2},1)\;\text{for}\;l>1, \end{array}$$

where $\omega_{pk}∼\text{Gam}(\kappa /2,\kappa /2)$ is called local shrinkage, and $\theta_{k}=\prod_{l=1}^{k}\delta_{l}$ is called global shrinkage, imposing increasingly strong shrinkage on higher‐indexed factors. This hierarchical shrinkage structure penalizes higher‐indexed factors more strongly.

Default hyperparameters are κ=3, $a_{1}=2.1$, and $a_{2}=3.1$. For the residual variances, we assume $\psi_{p}∼\text{Inv‐Gamma}(1,0.3)$. This choice are based on the guidelines by Bhattacharya and Dunson [45] and Durante [50].

##### 2.2.1.3 Estimation

The parameters in the Stack FA are typically estimated using Gibbs sampling.

To choose K, the standard procedure is the application of the eigenvalue decomposition (EVD) to the covariance matrices and retain the number of factors explaining a threshold level of explained variance (e.g., ≥ 5%). This is a *post‐hoc* strategy recommended by BMSFA. We then run the model again with the estimated numbers of factors.

Alternatively, Bhattacharya and Dunson proposed a dynamic truncation strategy, in which K is initialized to 3log(P) and adaptively pruned or expanded during sampling. This approach is implemented in MATLAB.

#### 2.2.2 Ind FA

##### 2.2.2.1 Model

An alternative approach is to fit separate FA models for each study (Ind FA). Specifically, for study s we model

(8) $$\underset{\left(P\times 1\right)}{\underbrace{y_{is}}}=\underset{\left(P\times J_{s}\right)}{\underbrace{\Lambda_{s}}}\;\underset{\left(J_{s}\times 1\right)}{\underbrace{l_{is}}}+\underset{\left(P\times 1\right)}{\underbrace{\epsilon_{is}}},$$

where $\Lambda_{s}$ is the $P\times J_{s}$ loadings matrix specific to study s, $l_{is}∼\text{MVN}(0,I_{J_{s}})$, and $\epsilon_{is}∼\text{MVN}(0,\Psi_{s})$ with $\Psi_{s}=\text{diag}(\psi_{1s},\ldots ,\psi_{Ps})$. The marginal covariance of $y_{is}$ is then:

(9) $$\sum_{s}=\Lambda_{s}\Lambda_{s}^{⊤}+\Psi_{s},$$

which we refer to as the study‐specific covariance.

Under Ind FA, any signals discovered in one study are not necessarily reproducible in the others, as Ind FA does not pool information across studies.

##### 2.2.2.2 Priors

We again adopt the MGPS prior, now applied independently to each $\Lambda_{s}$:

(10) $$\lambda_{pk}^{\left(s\right)}∼N(0,\omega_{pk}^{(s),-1}\theta_{k}^{(s),-1}),$$

with analogous priors on $\omega_{pk}^{\left(s\right)}$ and $\theta_{k}^{\left(s\right)}$, that is, $\omega_{pk}^{(s),-1}∼\text{Gamma}(\kappa /2,\kappa /2)$, and $\theta_{k}^{(s),-1}=\prod_{l=1}^{k}\delta_{l}^{\left(s\right)}$.

##### 2.2.2.3 Estimation

Each study is fit separately via Gibbs sampling.

The number of factors $J_{s}$ can be selected using the same strategies as in Stack FA—either through eigenvalue decomposition or adaptive truncation schemes.

### 2.3 Advanced Integrative Factor Approach

While Stack FA and Ind FA provide simple strategies for multi‐study analysis, they fail to exploit shared structure across studies or to model study‐specific variations in a unified framework. To overcome these limitations, several advanced Bayesian integrative factor models have been developed. These models allow for joint modeling of common and study‐specific variation in a statistically principled way.

#### 2.3.1 PFA

##### 2.3.1.1 Model

Perturbed Factor Analysis (PFA) [33] builds on the Stack FA model (Equation (1)) by introducing study‐specific perturbation matrices $Q_{s}$ that account for deviations from a shared loading structure. The model is:

(11) $$\begin{array}{ll} \underset{\left(P\times P\right)}{\underbrace{Q_{s}}}\;\underset{\left(P\times 1\right)}{\underbrace{y_{is}}} & =\underset{\left(P\times K\right)}{\underbrace{\Phi }}\;\underset{\left(K\times 1\right)}{\underbrace{f_{is}}}+\underset{\left(P\times 1\right)}{\underbrace{\epsilon_{is}}}, \end{array}$$

where $Q_{s}\in {\mathbb{R}}^{P\times P}$ is a transformation matrix for study s, with $Q_{1}=I_{P}$ fixed for a reference study. For s>1, $Q_{s}∼\text{MN}(I_{P},\alpha_{Q}I_{P},\alpha_{Q}I_{P})$ follows a matrix normal distribution centered at the identity matrix with row covariance and column covariance being $\alpha_{Q}$ times the identity matrix. Thus $\alpha_{Q}$ is a scale parameter that sets how much $Q_{s}$ can deviate from the identity, controlling the amount of perturbation away from the reference study.

Factor scores are modeled as $f_{is}∼\text{MVN}(0,V)$, where $V=\text{diag}(\nu_{1},\ldots ,\nu_{K})$. Unlike simpler FA models that might fix $V=I_{K}$, here the heteroscedastic variances $\nu_{k}$ help address the rotation identifiability issue. We can equivalently view $\Phi V^{1/2}$ as the common loadings matrix shared by all studies. The residual term $\epsilon_{is}∼\text{MVN}(0,\Psi )$, with $\Psi =\text{diag}(\psi_{1},\ldots ,\psi_{P})$ shared across studies.

In PFA, the marginal covariance of $y_{is}$ for each study is: $\sum_{s}=Q_{s}^{-1}(\Phi V\Phi^{⊤}+\Psi ){\left(Q_{s}^{-1}\right)}^{⊤}$, with the common covariance defined as $\sum_{\Phi }=\Phi V\Phi^{⊤}+\Psi$. Note that for s=1, $\sum_{1}=\sum_{\Phi }$ since $Q_{1}=I_{P}$. Study‐specific covariances are then defined as $\sum_{\Lambda_{s}}=\sum_{s}-\sum_{\Phi }$.

##### 2.3.1.2 Priors

PFA uses the same MGPS prior for Φ as Stack FA and Ind FA. The variance components $\nu_{k}$ have inverse‐gamma priors: $\nu_{k}∼\text{IG}(a_{v},b_{v})$, with the default values $a_{\nu }=10$ and $b_{\nu }=0.1$. The residual variances are assumed to have a weakly informative inverse‐gamma prior $\psi_{p}∼\text{IG}(0.1,0.1)$ are weakly informative. The perturbation scale $\alpha_{Q}$ also follows an inverse‐gamma prior: $\alpha_{Q}∼\text{IG}(0.1,0.1)$.

##### 2.3.1.3 Estimation

All parameters are estimated via MCMC with a Gibbs sampling algorithm. The parameter $\alpha_{Q}$ for the perturbation matrix can either be sampled from its inverse‐gamma posterior or specified by the user. Due to the presence of $Q_{s}$, inference in PFA is more computationally intensive than in Stack FA or Ind FA. However, it offers the critical advantage of aligning each study to a common latent structure, thus preserving global patterns while accounting for structured study‐specific variations.

To determine the number of factors, PFA employs the adaptive truncation procedure introduced by Bhattacharya and Dunson [45]. At each iteration, the algorithm imputes the mean absolute value of the loadings in each column of Φ. If all the loading for a given factor falls below a predefined cutoff value (e.g., $10^{-3}$), that column is removed from the model. As a result, the number of factors decreases monotonically throughout the sampling process, allowing the model complexity to be gradually pruned.

When the cutoff is appropriately chosen and sufficient burn‐in is used, this adaptive truncation typically stabilizes early in sampling. However, convergence to a single value is not guaranteed. In practice, the number of factors may still fluctuate slightly in the latter part of the chain, necessitating post‐processing.

#### 2.3.2 MOM‐SS

##### 2.3.2.1 Model

The MOM‐SS model extends the Stack FA model by incorporating study‐specific intercepts and noise to correct for both additive and multiplicative batch effects, as well as a regression adjustment. The model is formulated as:

(12) $$\underset{\left(P\times 1\right)}{\underbrace{y_{is}}}=\underset{\left(P\times 1\right)}{\underbrace{\alpha_{s}}}+\underset{\left(P\times Q\right)}{\underbrace{\beta }}\;\underset{\left(Q\times 1\right)}{\underbrace{x_{is}}}+\underset{\left(P\times K\right)}{\underbrace{\Phi }}\;\underset{\left(K\times 1\right)}{\underbrace{f_{is}}}+\underset{\left(P\times 1\right)}{\underbrace{\epsilon_{is}}},$$

where $\alpha_{s}\in {\mathbb{R}}^{P}$ is a study‐specific intercept vector, $x_{is}\in {\mathbb{R}}^{Q}$ is a vector of covariates, $\beta \in {\mathbb{R}}^{P\times Q}$ is the matrix of regression coefficients, $\Phi \in {\mathbb{R}}^{P\times K}$ is the common loading matrix, and $f_{is}∼\text{MVN}(0,I_{K})$ are the latent factors. The error term $\epsilon_{is}∼\text{MVN}(0,\Psi_{s})$ accounts for study‐specific noise with $\Psi_{s}=\text{diag}(\psi_{1s},\ldots ,\psi_{Ps})$.

A matrix form of the model (12) stacks all observations across studies:

(13) $$\begin{array}{cc} \underset{\left(N\times P\right)}{\underbrace{Y}} & =\underset{\left(N\times S\right)}{\underbrace{M}}\;\underset{\left(S\times P\right)}{\underbrace{\alpha^{⊤}}}+\underset{\left(N\times Q\right)}{\underbrace{X}}\;\underset{\left(Q\times P\right)}{\underbrace{\beta^{⊤}}}\\ & \;\;+\underset{\left(N\times K\right)}{\underbrace{F}}\;\underset{\left(K\times P\right)}{\underbrace{\Phi^{⊤}}}+\underset{\left(N\times P\right)}{\underbrace{E}},\\ \text{with}\;M & =\left[\begin{array}{cccc} 1_{N_{1}} & 0 & \cdots & 0\\ 0 & 1_{N_{2}} & \cdots & 0\\ \vdots & \vdots & \ddots & \vdots \\ 0 & 0 & \cdots & 1_{N_{S}} \end{array}\right],\;\text{and}\;\alpha =\left[\begin{array}{cccc} \alpha_{11} & \alpha_{12} & \cdots & \alpha_{1S}\\ \alpha_{21} & \alpha_{22} & \cdots & \alpha_{2S}\\ \vdots & \vdots & \ddots & \vdots \\ \alpha_{P1} & \alpha_{P2} & \cdots & \alpha_{PS} \end{array}\right], \end{array}$$

where M is a block‐diagonal matrix encoding group/study membership, α contains intercepts for each study and variable, X is the matrix of observed covariates, F contains latent factors, and E contains residuals. The marginal covariance in study s is $\sum_{s}=(\Phi \Phi^{⊤}+\Psi_{s})$.

##### 2.3.2.2 Priors

MOM‐SS adopts a moment‐based non‐local spike‐and‐slab (NLP) by Johnson et al. [51, 52] on the entries of Φ to induce sparsity in the loadings. Each loading $\phi_{pk}$ is modeled by

(14) $$\begin{array}{cc} \phi_{pk} & ∼(1-\gamma_{pk})f_{\text{spike}}+\gamma_{pk}f_{\text{slab}},\\ \gamma_{pk} & ∼\text{Bernoulli}(\zeta_{k}), \end{array}$$

where the **spike** is a normal density with a small variance $f_{\text{spike}}=𝒩(0,\tau_{0})$, and the **slab** is a moment‐penalized normal density, which takes the form $f_{\text{slab}}(\phi_{pk})=(\phi_{pk}^{2}/\tau_{1})𝒩(0,\tau_{1})$. Hence, the prior assigns zero density at $\phi_{pk}$ whenever $\gamma_{pk}=1$, so that $\phi_{pk}$ is forced away from zero (the non‐local property). The default values for $\tau_{0}$ and $\tau_{1}$ are equal to 0.026 and 0.28 to distinguish practically correlated factors. The inclusion probability $\zeta_{k}∼\text{Beta}(a_{\zeta }/k,b_{\zeta })$ controls the proportion of active loadings, with defaults $a_{\zeta }=b_{\zeta }=1$.

Priors for the intercepts $\alpha_{s}$ and regression coefficients β follow a standard normal distribution: $𝒩(0,\sigma_{\text{reg}}^{2})$ with default $\sigma_{\text{reg}}^{2}=1$. Each residual variance $\psi_{ps}$ follows an inverse‐gamma prior: $\psi_{ps}∼\text{IG}(0.5,0.5)$.

##### 2.3.2.3 Estimations

Parameter estimation is performed using the Expectation‐Maximization (EM) algorithm, which consists of two iterative steps. The E‐step computes the conditional expectations of the latent factors and the M‐step maximizes the complete‐data log‐posterior, updating $\alpha_{s}$, β, Φ, and $\Psi_{s}$. A coordinate descent step is embedded in the M‐step to accommodate the non‐local slab structure.

To initialize parameters, two approaches based on least‐square estimation and eigenvalue decomposition are provided—one using eigenvalue decomposition with varimax rotation and another without. Ten‐fold cross‐validation is used to select the best initialization.

After estimation, the effective number of factors is determined by discarding columns of Φ with low posterior inclusion probabilities (i.e., $\gamma_{pk}\approx 0$). Factors are reordered *post hoc* based on the number of non‐zero loadings to enhance interpretability.

#### 2.3.3 SUFA

##### 2.3.3.1 Model

SUbspace Factor Analysis (SUFA) [35] explicitly models both shared and study‐specific variation by augmenting a shared factor structure with study‐specific subspaces. The model is defined as:

(15) $$\underset{\left(P\times 1\right)}{\underbrace{y_{is}}}=\underset{\left(P\times K\right)}{\underbrace{\Phi }}\;\underset{\left(K\times 1\right)}{\underbrace{f_{is}}}+\underset{\left(P\times K\right)}{\underbrace{\Phi }}\;\underset{\left(K\times J_{s}\right)}{\underbrace{A_{s}}}\;\underset{\left(J_{s}\times 1\right)}{\underbrace{l_{is}}}+\underset{\left(P\times 1\right)}{\underbrace{\epsilon_{is}}},$$

where $\Phi \in {\mathbb{R}}^{P\times K}$ is a shared loading matrix, $fis∼\text{MVN}(0,I_{K})$ are the corresponding common latent factors, $A_{s}\in {\mathbb{R}}^{K\times J_{s}}$ is a transformation matrix for study‐specific components, and $l_{is}∼\text{MVN}(0,I_{J_{s}})$ are study‐specific latent factors. The residuals $\epsilon_{is}∼\text{MVN}(0,\Psi )$ are shared across studies, with $\Psi =\text{diag}(\psi_{1},\ldots ,\psi_{P})$. SUFA decomposes the total variability into (i) a shared low‐dimensional subspace common to all studies and (ii) study‐specific subspaces that explain extra variation unique to each study. The first term $\Phi f_{is}$ is the common factor effect shared across studies, while $\Phi A_{s}l_{is}$ introduces additional latent dimensions unique to the study s. In this way, SUFA ensures all study‐specific loadings $\Lambda_{s}=\Phi A_{s}$ lie in the column space of Φ. Identifiability is guaranteed by enforcing $\sum_{s}J_{s}\leq K$. The residuals $\epsilon_{is}$ follow MVN(0,Ψ) with $\Psi =\text{diag}(\psi_{1},\ldots ,\psi_{P})$ invariant across studies.

The marginal covariance of the study s is $\sum_{s}=\Phi \Phi^{⊤}+\Phi A_{s}A_{s}^{⊤}\Phi^{⊤}+\Psi$, where $\sum_{\Phi }=\Phi \Phi^{⊤}$ represents the shared variance, while $\sum_{\Lambda_{s}}=\Lambda_{s}\Lambda_{s}^{⊤}=\Phi A_{s}A_{s}^{⊤}\Phi^{⊤}$ captures study‐specific effects in study s.

##### 2.3.3.2 Priors

To encourage sparsity in the shared loading matrix Φ, SUFA adopts a Dirichlet‐Laplace (DL) [53] prior. The DL prior combines computational efficiency with strong theoretical properties, such as achieving near‐minimax optimal posterior contraction rates in high‐dimensional settings [54]. Formally, the vectorized loading matrix vec(Φ) follows $\text{DL}(a_{DL})$ distribution, defined as:

(16) $$\begin{array}{cc} \phi_{pk} & ∼\text{Laplace}(\omega_{p,DL}\theta_{DL}),\\ \omega_{DL} & ∼\text{Dir}(a_{DL},\cdots \;,a_{DL}),\theta_{DL}∼\text{Gam}(a_{DL}P,0.5), \end{array}$$

where $\phi_{pk}$ denotes the (p,k)th element of Φ. Each scale parameter $\omega_{p,DL}$ comes from a Dirichlet distribution, controlling local shrinkage across rows, while $\theta_{DL}$ is a global shrinkage parameter drawn from a Gamma distribution. The hyperparameter $a_{DL}$ regulates overall sparsity and is typically set to 0.5 by default.

For the study‐specific transformation matrices $A_{s}$, SUFA uses independent Gaussian priors: $a_{pjs}∼𝒩(0,\sigma_{A}^{2})$, where $\sigma_{A}^{2}=1$ by default. These priors do not induce shrinkage, but help prevent information switching between elements.

The residual variances Ψ have log‐normal priors, that is, $\psi_{p}∼\log 𝒩(\mu_{\Psi },\sigma_{\Psi }^{2})$, which are known to improve numerical stability and mixing in hierarchical models [55]. By default, the hyperparameters $\mu_{\Psi }$ and $\sigma_{\Psi }^{2}$ are chosen such that $𝔼(\psi_{p})=1$ and $\mathrm{var}(\psi_{p})=7$ for p=1,…,P.

##### 2.3.3.3 Estimation

SUFA employs a hybrid MCMC approach, combining a Hamiltonian Monte Carlo (HMC) sampler [56] within a Gibbs sampling framework. Rather than sampling from the full joint likelihood, SUFA marginalizes over the latent factors and conducts inference using the marginal likelihood. In each iteration, the parameters, that is, Φ, Ψ, and $A_{s}$, s=1,…,S, are updated via the HMC sampler, while the DL hyperparameters are sampled from their conditional distributions using standard Gibbs steps. SUFA parallelizes the HMC step across studies to improve computational efficiency. However, this comes at the cost of additional gradient computations, with runtime scaling approximately quadratically in the number of variables P.

For model selection, SUFA requires pre‐specification of a maximum value for the number of shared factors K. To prune the K into a desired number, it uses a singular value decomposition (SVD)‐based algorithm known as *Implicitly Restarted Lanczos Bidiagonalization* [57], selecting the smallest K that explains at least 95% of the total variability before running the MCMC algorithm. To specify the number of study‐specific factors $J_{s}$, SUFA provides a default heuristic that sets $J_{s}=K/S$, evenly distributing the total latent component among studies.

#### 2.3.4 BMSFA

##### 2.3.4.1 Model

Bayesian Multi‐Study Factor Analysis (BMSFA) extends the classical factor model by simultaneously modeling shared and study‐specific structures across multiple datasets. Initially proposed in a frequentist framework [29], a fully Bayesian implementation was later introduced [30]. The BMSFA model is defined as:

(17) $$\begin{array}{c} \underset{\left(P\times 1\right)}{\underbrace{y_{is}}}=\underset{\left(P\times K\right)}{\underbrace{\Phi }}\;\underset{\left(K\times 1\right)}{\underbrace{f_{is}}}+\underset{\left(P\times J_{s}\right)}{\underbrace{\Lambda_{s}}}\;\underset{\left(J_{s}\times 1\right)}{\underbrace{l_{is}}}\;\underset{\left(P\times 1\right)}{\underbrace{\epsilon_{is}}}, \end{array}$$

where $\Phi \in {\mathbb{R}}^{P\times K}$ is the common loading matrix shared by all studies, $f_{is}∼\text{MVN}(0,I_{K})$ are the corresponding shared latent factors, $\Lambda_{s}\in {\mathbb{R}}^{P\times J_{s}}$ is the study‐specific loading matrix for study s, and $l_{is}∼\text{MVN}(0,I_{J_{s}})$ are the study‐specific latent factors. The residual term $\epsilon_{is}∼\text{MVN}(0,\Psi_{s})$, with $\Psi_{s}=\text{diag}(\psi_{1s},\ldots ,\psi_{Ps})$.

Under this formulation, the marginal covariance for study s is: $\sum_{s}=\Phi \Phi^{⊤}+\Lambda_{s}\Lambda_{s}^{⊤}+\Psi_{s},$ where $\Phi \Phi^{⊤}$ represents the shared covariance matrix, and each $\Lambda_{s}\Lambda_{s}^{⊤}$ captures additional variability unique to study s. This dual structure generalizes both Stack FA and Ind FA, allowing for modeling heterogeneity across studies.

##### 2.3.4.2 Priors

BMSFA applies the Multiplicative Gamma Process Shrinkage (MGPS) prior [45] to both the shared loading matrix Φ and the study‐specific loading matrices $\Lambda_{s}$, encouraging shrinkage. Each loading $\phi_{pk}$ (or $\lambda_{pj}^{\left(s\right)}$) is assigned a normal prior with variance governed by local and column‐wise shrinkage parameters, as described in Equation (7). This setup ensures that higher‐indexed columns are increasingly shrunk toward zero. Residual variances $\psi_{ps}$ are modeled independently across studies, using inverse‐gamma priors: $\psi_{ps}∼\text{IG}(a_{\psi },b_{\psi })$, with default values typically set to (1,0.3).

##### 2.3.4.3 Estimations

BMSFA is estimated using a Gibbs sampling algorithm, with full conditional updates for all model parameters. To obtain the posterior means or medians of Φ and $\Lambda_{s}$, the authors recommend either Orthogonal Procrustes (OP) rotation or spectral decomposition (SD) for aligning MCMC draws and improving interpretability.

To determine the number of factors, one can run the sampler with large initial values of K and $J_{s}$, then post‐process the posterior mean of $\sum_{s}$ using an eigenvalue decomposition, retaining factors that explain a specified fraction of variance [30]. To determine the number of factors, BMSFA initializes with large values for K and $J_{s}$, and then post‐processes the posterior samples of the shared covariance matrix, $\Phi \Phi^{⊤}$, and study‐specific covariance matrix, $\Lambda_{s}\Lambda_{s}^{⊤}$, using eigenvalue decomposition. Factors are retained if their associated eigenvalues explain a sufficiently large portion of the variance (e.g., 5%), following the strategy described in De Vito et al. [30].

#### 2.3.5 CAVI

##### 2.3.5.1 Model and Priors

The Coordinate‐Ascent Variational Inference (CAVI) approach [31] is applied to the same BMSFA model introduced above, that is, the shared/study‐specific factor decomposition and the corresponding conjugate priors on loading matrices and residual variances are unchanged. Hence, the distinguishing feature of CAVI is not the statistical model, but the inference strategy used to approximate the posterior distribution.

##### 2.3.5.2 Estimation

CAVI fits the BMSFA model via variational inference, which replaces the intractable posterior with a tractable approximation drawn from a restricted family [58, 59]. The approximation is selected by minimizing the Kullback–Leibler divergence to the true posterior, equivalently by maximizing the evidence lower bound (ELBO). The ELBO balances (i) a term encouraging good fit to the observed data under the joint model and (ii) a regularization term that discourages approximating distributions that diverge from the prior distribution.

A standard choice is the mean‐field variational family, which factorizes the approximation across blocks of parameters. Under this assumption, CAVI proceeds by iteratively updating each variational factor while holding the others fixed, setting it to its coordinate‐wise optimal form computed from the model's complete conditional distribution averaged with respect to the current approximation of the remaining parameters. Repeating these coordinate‐wise updates until convergence yields the Coordinate‐Ascent Variational Inference (CAVI) algorithm.

#### 2.3.6 BLAST

##### 2.3.6.1 Model

The Bayesian Latent Analysis through Spectral Training (BLAST) [32] is equivalent to the BMSFA specification introduced above. For each study s=1,…,S, let $Y_{s}\in {\mathbb{R}}^{n_{s}\times P}$ denote the data matrix, $F_{s}\in {\mathbb{R}}^{n_{s}\times K}$ the shared factor scores, and $L_{s}\in {\mathbb{R}}^{n_{s}\times J_{s}}$ the study‐specific factor scores. Define the combined factor score matrix $M_{s}=[F_{s}\;L_{s}]\in {\mathbb{R}}^{n_{s}\times (K+J_{s})}$ and the combined loading matrix $\Lambda_{s}^{\text{all}}=[\Phi \;\Lambda_{s}]\in {\mathbb{R}}^{P\times (K+J_{s})}$. Then BLAST can be written as

$$Y_{s}=M_{s}{\left(\Lambda_{s}^{\text{all}}\right)}^{⊤}+E_{s},$$

which explicitly separates the shared (Φ) and study‐specific ($\Lambda_{s}$) loading components.

##### 2.3.6.2 Priors

BLAST places conjugate Normal–Inverse‐Gamma priors on the rows of the loading matrices and on the corresponding residual variances. For j=1,…,P, let $\phi_{j}^{⊤}$ denote the j‐th row of the shared loading matrix Φ:

$$\phi_{j}|\psi_{j}∼{𝒩}_{K}\;\left(0,\;\tau_{\Phi }^{2}\;\psi_{j}\;I_{K}\right),\;\psi_{j}∼\text{IG}\;\left(\frac{\nu_{0}}{2},\;\frac{\nu_{0}\psi_{0}}{2}\right).$$

Similarly, for each study s and j=1,…,P, let $\lambda_{js}^{⊤}$ denote the jth row of the study‐specific loading matrix $\Lambda_{s}$:

$$\lambda_{js}|\psi_{js}∼{𝒩}_{J_{s}}\;\left(0,\;\tau_{\Lambda }^{2}\;\psi_{js}\;I_{J_{s}}\right),\;\psi_{js}∼\text{IG}\;\left(\frac{\nu_{0}}{2},\;\frac{\nu_{0}\psi_{0}}{2}\right).$$

##### 2.3.6.3 Estimation

Conditionally on the latent factors, the loading matrices are updated through Gaussian regression steps with diagonal residual covariance. Let $n=\sum_{s=1}^{S}n_{s}$, stack the data as $Y={\left[Y_{1}^{⊤},\ldots ,Y_{S}^{⊤}\right]}^{⊤}\in {\mathbb{R}}^{n\times P}$, and the shared factors as $F={\left[F_{1}^{⊤},\ldots ,F_{S}^{⊤}\right]}^{⊤}\in {\mathbb{R}}^{n\times K}$.

*Shared loadings*. Given F, the shared loading matrix $\Phi \in {\mathbb{R}}^{P\times K}$ is inferred from the surrogate regression

$$\begin{array}{cc} Y^{c} & =F\;\Phi^{⊤}+E,\;\text{vec}(E)∼{𝒩}_{nP}\;\left(0,\;\sum ⊗I_{n}\right),\\ \sum & =\text{diag}(\psi_{1},\ldots ,\psi_{P}), \end{array}$$

where $Y^{c}$ denotes the projected/constructed response used in the surrogate regression.

*Study‐specific loadings*. For each study s, after removing the shared component, define the residual matrix

$$R_{s}=Y_{s}-F_{s}\Phi^{⊤}\in {\mathbb{R}}^{n_{s}\times P}.$$

Given $L_{s}\in {\mathbb{R}}^{n_{s}\times J_{s}}$, the study‐specific loading matrix $\Lambda_{s}\in {\mathbb{R}}^{P\times J_{s}}$ is inferred from

$$\begin{array}{ll} R_{s} & =L_{s}\;\Lambda_{s}^{⊤}+E_{s},\;\text{vec}(E_{s})∼{𝒩}_{n_{s}P}\left(0,\;\sum_{s}⊗I_{n_{s}}\right),\;\\ \sum_{s} & =\text{diag}(\psi_{1s},\ldots ,\psi_{Ps}). \end{array}$$

#### 2.3.7 Tetris

##### 2.3.7.1 Model

Tetris [60] generalizes BMSFA by enabling the combination of shared, partially shared, and study‐specific latent factors across different sets of studies. This is achieved through the introduction of a binary indicator matrix $𝒯\in {0,1}^{S\times K^{*}}$, where each row corresponds to a study and each column indicates whether a given factor is active in that study. The model is written as:

(18) $$\underset{\left(P\times 1\right)}{\underbrace{y_{is}}}=\underset{\left(P\times K^{*}\right)}{\underbrace{\Phi^{*}}}\;\underset{\left(K^{*}\times K^{*}\right)}{\underbrace{T_{s}}}\;\underset{\left(K^{*}\times 1\right)}{\underbrace{f_{is}}}+\underset{\left(P\times 1\right)}{\underbrace{\epsilon_{is}}},$$

where $\Phi^{*}\in {\mathbb{R}}^{P\times K}$ is the loading matrix, $T_{s}\in {0,1}^{K^{\times }K}$ is a diagonal matrix selecting factors for study s, $f_{is}∼\text{MVN}(0,I_{K})$, and $\epsilon_{is}∼\text{MVN}(0,\Psi_{s})$ with $\Psi_{s}=\text{diag}(\psi_{1s},\ldots ,\psi_{Ps})$.

The binary matrix 𝒯 governs the structure of $T_{s}$ for each study. Each row of 𝒯 corresponds to a study, and each column corresponds to a latent factor. A factor is considered: (1) common if the corresponding column of 𝒯 contains all 1s, (2) study‐specific, if the column contains a 1 in only one row, (3) partially shared if only some rows contain 1s.

To illustrate, consider a case with S=3 studies. Suppose that the model estimates the following binary matrices $T_{1}$, $T_{2}$, $T_{3}$, and 𝒯:

$$\begin{array}{ll} T_{1} & =\left[\begin{array}{cccc} 1 & 0 & 0 & 0\\ 0 & 1 & 0 & 0\\ 0 & 0 & 1 & 0\\ 0 & 0 & 0 & 0 \end{array}\right],\;T_{2}=\left[\begin{array}{cccc} 1 & 0 & 0 & 0\\ 0 & 1 & 0 & 0\\ 0 & 0 & 0 & 0\\ 0 & 0 & 0 & 1 \end{array}\right],\\ T_{3} & =\left[\begin{array}{cccc} 1 & 0 & 0 & 0\\ 0 & 0 & 0 & 0\\ 0 & 0 & 0 & 0\\ 0 & 0 & 0 & 0 \end{array}\right],\;𝒯=\left[\begin{array}{cccc} 1 & 1 & 1 & 0\\ 1 & 1 & 0 & 1\\ 1 & 0 & 0 & 0 \end{array}\right], \end{array}$$

In this example, Factor 1 is common to all studies (column of all 1s), Factor 2 is shared between studies 1 and 2, Factor 3 is specific to study 1, and Factor 4 is specific to study 2.

The common loadings and study‐specific loadings can be extracted from $\Phi^{*}$ by manipulating the $T_{s}$ matrices. We can define $P\in {0,1}^{K^{*}\times K^{*}}$ a diagonal matrix with 1 in the kth row if column k in 𝒯 has all 1s. Then the common loadings matrices are $\Phi =\Phi^{*}P$. For each study s, let $R_{s}=T_{s}-P$, then $\Lambda_{s}=\Phi^{*}R_{s}$ are the loadings corresponding to the partially shared or study‐specific factors used by that study. The marginal covariance for the study s is: $\sum_{s}=\Phi^{*}T_{s}{\Phi^{*}}^{⊤}+\Psi_{s}=\Phi^{*}P{\Phi^{*}}^{⊤}+\Phi^{*}R_{s}{\Phi^{*}}^{⊤}+\Psi_{s}$. The common covariance component is extracted by $\Phi^{*}P{\Phi^{*}}^{⊤}$, and the study‐specific covariance is $\Phi^{*}R_{s}{\Phi^{*}}^{⊤}$.

##### 2.3.7.2 Priors

Tetris places MGPS priors on $\Phi^{*}$ to encourage shrinkage and reduce overfitting. For the factor indicator matrix, i.e., 𝒯, it adopts an Indian Buffet Process (IBP) prior [61], a nonparametric prior well‐suited for modeling latent binary matrices with potentially infinite columns. The IBP is controlled by hyperparameters $\alpha_{𝒯}$ and $\beta_{𝒯}$, which regulate the expected number of active factors and their distribution across studies. By default, $\alpha_{𝒯}=1.25\times S$ and $\beta_{𝒯}=1$.

Each residual variance $\psi_{ps}$ again follows an inverse‐gamma prior, with hyperparameters set as in BMSFA.

##### 2.3.7.3 Estimation

Tetris uses a Metropolis‐within‐Gibbs sampler [62]. The factor allocation matrix 𝒯 is updated using a Metropolis–Hastings step, while all other parameters (including $\Phi^{*}$, $\Psi_{s}$, and latent factors) are sampled using the Gibbs sampler.

Once the MCMC chains of 𝒯 are obtained, the point estimate of 𝒯 is selected by identifying the matrix that lies in the mode of the posterior—specifically, the configuration with the highest local density under a neighborhood metric. Conditional on the selected 𝒯, the model is re‐fit using standard Gibbs sampling to obtain aligned posterior samples of factors and loadings with consistent dimensions.

The number of factors is computed via the estimated 𝒯 matrix. The number of common factors K is the number of columns containing all 1s in 𝒯, while the number of study‐specific factors for study s refers to the number of 1s in the sth row in 𝒯, subtracting by K.

In summary, Table 1 summarizes the method we discussed above and its priors.

**TABLE 1:** Summary of the Bayesian integrative factor analysis models considered in this study, including their prior assumptions and structural specifications.

Model	Model formula	Covariance decomposition	Priors
Stack FA	$\begin{array}{ll} y_{is} & =\Phi f_{is}+\epsilon_{is}\\ f_{is} & ∼\text{MVN}(0,I_{K})\\ \epsilon_{is} & ∼\text{MVN}(0,\Psi )\\ \Psi & =\text{diag}(\psi_{1},\ldots ,\psi_{P}) \end{array}$	$\begin{array}{ll} & \text{Marginal covariance:}\\ & \;\sum_{s}=\Phi \Phi^{⊤}+\Psi \\ & \text{Common covariance:}\\ & \;\sum_{\Phi }=\Phi \Phi^{⊤} \end{array}$	$\begin{array}{ll} \text{For}\; & \Phi :\\ & \phi_{pk}|\omega_{pk},\theta ∼𝒩(0,\omega_{pk}^{-1}\theta_{k}^{-1})\\ & \omega_{pk}∼\text{Gam}(\kappa /2,\kappa /2)\\ & \theta_{k}=\prod_{l=1}^{k}\delta_{l},\;\delta_{1}∼\text{Gam}(a_{1},1),\\ & \delta_{l}∼\text{Gam}(a_{2},1),\;\text{for}\;l>1\\ \text{For}\; & \Psi :\\ & \psi_{p}∼\text{IG}(a_{\psi },b_{\psi }) \end{array}$
Ind FA	$\begin{array}{ll} y_{is} & =\Lambda_{s}l_{is}+\epsilon_{is}\\ l_{is} & ∼\text{MVN}(0,I_{J_{s}})\\ \epsilon_{is} & ∼\text{MVN}(0,\Psi_{s})\\ \Psi_{s} & =\text{diag}(\psi_{1s},\psi_{2s},\ldots ,\psi_{Ps}) \end{array}$	$\begin{array}{ll} & \text{Marginal covariance:}\\ & \;\sum_{s}=\Lambda_{s}\Lambda_{s}^{⊤}+\Psi_{s}\\ & \text{Study‐specific covariance:}\\ & \;\sum_{\Phi }=\Lambda_{s}\Lambda_{s}^{⊤} \end{array}$	$\begin{array}{ll} \text{For}\; & \Lambda_{s}:\\ & \lambda_{pm}|\omega_{pm},\theta_{m}∼𝒩(0,\omega_{pm}^{-1}\theta_{m}^{-1})\\ & \omega_{pm}∼\text{Gam}(\kappa^{s}/2,\kappa^{s}/2)\\ & \theta_{m}=\prod_{l=1}^{m}\delta_{l},\;\delta_{1}∼\text{Gam}(a_{1}^{s},1),\\ & \delta_{l}∼\text{Gam}(a_{2}^{s},1),\;\text{for}\;l>1\\ \text{For}\; & \Psi_{s}:\\ & \psi_{ps}∼\text{IG}(a_{\psi },b_{\psi }) \end{array}$
PFA	$\begin{array}{ll} Q_{s} & y_{is}=\Phi f_{is}+\epsilon_{is}\\ Q_{s} & ∼{\text{MN}}_{P\times P}(I_{P},\alpha_{Q}I_{P},\alpha_{Q}I_{P}),\\ \; & Q_{1}=I_{P}\\ f_{is} & ∼\text{MVN}(0,V)\\ V & =\text{diag}(\nu_{1},\nu_{2},\ldots ,\nu_{K})\\ \epsilon_{is} & ∼\text{MVN}(0,\Psi )\\ \Psi & =\text{diag}(\psi_{1},\psi_{2},\ldots ,\psi_{P}) \end{array}$	$\begin{array}{ll} & \text{Marginal covariance:}\\ & \;\sum_{s}=Q_{s}^{-1}(\Phi V\Phi^{⊤}+\Psi ){\left(Q_{s}^{-1}\right)}^{⊤}\\ & \text{Common covariance:}\\ & \;\sum_{\Phi }=\Phi V\Phi^{⊤}+\Psi \\ & \text{Study‐specific covariance:}\\ & \;\sum_{\Lambda_{s}}=\sum_{s}-\sum_{\Phi } \end{array}$	$\begin{array}{ll} \text{For}\; & \Phi :\\ & \phi_{pk}|\omega_{pk},\theta ∼𝒩(0,\omega_{pk}^{-1}\theta_{k}^{-1})\\ & \omega_{pk}∼\text{Gam}(\kappa /2,\kappa /2)\\ & \theta_{k}=\prod_{l=1}^{k}\delta_{l},\;\delta_{1}∼\text{Gam}(a_{1},1),\\ & \delta_{l}∼\text{Gam}(a_{2},1),\;\text{for}\;l>1\\ \text{For}\; & V\;\text{and}\;\Psi :\\ & v_{k}∼\text{IG}(a_{\nu },b_{\nu })\\ & \psi_{p}∼\text{IG}(a_{\psi },b_{\psi }) \end{array}$
MOM‐SS	$\begin{array}{ll} y_{is} & =\alpha_{s}+\beta x_{is}+\Phi f_{is}+\epsilon_{is}\\ f_{is} & ∼\text{MVN}(0,I_{K})\\ \epsilon_{is} & ∼\text{MVN}(0,\Psi_{s})\\ \Psi_{s} & =\text{diag}(\psi_{1s},\psi_{2s},\ldots ,\psi_{Ps}) \end{array}$	$\begin{array}{ll} & \text{Marginal covariance:}\\ & \;\sum_{s}=\Phi \Phi^{⊤}+\Psi_{s}\\ & \text{Common covariance:}\\ & \;\sum_{\Phi }=\Phi \Phi^{⊤} \end{array}$	$\begin{array}{ll} \text{For}\; & \Phi :\\ & \begin{array}{lll} \phi_{pk}|\gamma_{pk},\tau_{0},\tau_{1}= & (1-\gamma_{pk})f_{\text{spike}} & \\ & +\gamma_{pk}f_{\text{slab}} & \end{array}\\ & \gamma_{pk}∼\text{Bernoulli}(\zeta_{k})\\ & \zeta_{k}∼\text{Beta}(\frac{a_{\zeta }}{k},b_{\zeta })\\ & f_{\text{spike}}=𝒩(0,\tau_{0})\\ & f_{\text{slab}}=(\phi_{pk}^{2}/\tau_{1})𝒩(0,\tau_{1})\\ \text{For}\; & \alpha_{ps}\;\text{and}\;\beta_{p}:\\ & \alpha_{ps},\beta_{p}∼𝒩(0,\sigma_{\text{reg}}^{2}I)\\ \text{For}\; & \Psi_{s}:\\ & \psi_{ps}∼\text{IG}(a_{\psi },b_{\psi }) \end{array}$
SUFA	$\begin{array}{ll} y_{is} & =\Phi f_{is}+\Phi A_{s}l_{is}+\epsilon_{is}\\ f_{is} & ∼\text{MVN}(0,I_{K})\\ l_{is} & ∼\text{MVN}(0,I_{J_{s}})\\ \epsilon_{is} & ∼\text{MVN}(0,\Psi )\\ \Psi & =\text{diag}(\psi_{1},\psi_{2},\ldots ,\psi_{P}) \end{array}$	$\begin{array}{ll} & \text{Marginal covariance:}\\ & \;\sum_{s}=\Phi \Phi^{⊤}+\Phi A_{s}A_{s}^{⊤}\Phi^{⊤}+\Psi \\ & \text{Common covariance:}\\ & \;\sum_{\Phi }=\Phi \Phi^{⊤}+\Psi \\ & \text{Study‐specific covariance:}\\ & \;\sum_{\Lambda_{s}}=\Phi A_{s}A_{s}^{⊤}\Phi^{⊤} \end{array}$	$\begin{array}{ll} \text{For}\; & \Phi :\\ & \text{vec}(\Phi )∼\text{DL}(a_{DL})\\ \text{For}\; & A_{s}:\\ & a_{pjs}∼N(0,\sigma_{A}^{2})\\ \text{For}\; & \Psi :\\ & \psi_{p}∼\text{Log‐Normal}(\mu_{\psi },\sigma_{\psi }^{2}) \end{array}$
BMSFA	$\begin{array}{ll} y_{is} & =\Phi f_{is}+\Lambda_{s}l_{is}+\epsilon_{is}\\ f_{is} & ∼\text{MVN}(0,I_{K})\\ l_{is} & ∼\text{MVN}(0,I_{J_{s}})\\ \epsilon_{is} & ∼\text{MVN}(0,\Psi_{s})\\ \Psi_{s} & =\text{diag}(\psi_{1s},\psi_{2s},\ldots ,\psi_{Ps}) \end{array}$	$\begin{array}{ll} & \text{Marginal covariance:}\\ & \;\sum_{s}=\Phi \Phi^{⊤}+\Lambda_{s}\Lambda_{s}^{⊤}+\Psi_{s}\\ & \text{Common covariance:}\\ & \;\sum_{\Phi }=\Phi \Phi^{⊤}\\ & \text{Study‐specific covariance:}\\ & \;\sum_{\Lambda_{s}}=\Lambda_{s}\Lambda_{s}^{⊤} \end{array}$	$\begin{array}{ll} \text{For}\; & \Phi :\\ & \phi_{pk}|\omega_{pk},\theta ∼𝒩(0,\omega_{pk}^{-1}\theta_{k}^{-1})\\ & \omega_{pk}∼\text{Gam}(\kappa /2,\kappa /2)\\ & \theta_{k}=\prod_{l=1}^{k}\delta_{l},\;\delta_{1}∼\text{Gam}(a_{1},1),\\ & \delta_{l}∼\text{Gam}(a_{2},1),\;\text{for}\;l>1\\ \text{For}\; & \Lambda_{s}:\\ & \lambda_{pm}|\omega_{pm},\theta_{m}∼𝒩(0,\omega_{pm}^{-1}\theta_{m}^{-1})\\ & \omega_{pm}∼\text{Gam}(\kappa_{s}/2,\kappa_{s}/2)\\ & \theta_{m}=\prod_{l=1}^{m}\delta_{l},\;\delta_{1}∼\text{Gam}(a_{1}^{s},1),\\ & \delta_{l}∼\text{Gam}(a_{2}^{s},1),\;\text{for}\;l>1\\ \text{For}\; & \Psi_{s}:\\ & \psi_{ps}∼\text{IG}(a_{\psi },b_{\psi }) \end{array}$
BLAST	$\begin{array}{ll} y_{is} & =\Phi f_{is}+\Lambda_{s}l_{is}+\epsilon_{is}\\ f_{is} & ∼\text{MVN}(0,I_{K})\\ l_{is} & ∼\text{MVN}(0,I_{J_{s}})\\ \epsilon_{is} & ∼\text{MVN}(0,\Psi_{s})\\ \Psi_{s} & =\text{diag}(\psi_{1s},\psi_{2s},\ldots ,\psi_{Ps}) \end{array}$	$\begin{array}{ll} & \text{Marginal covariance:}\\ & \;\sum_{s}=\Phi \Phi^{⊤}+\Lambda_{s}\Lambda_{s}^{⊤}+\Psi_{s}\\ & \text{Common covariance:}\\ & \;\sum_{\Phi }=\Phi \Phi^{⊤}\\ & \text{Study‐specific covariance:}\\ & \;\sum_{\Lambda_{s}}=\Lambda_{s}\Lambda_{s}^{⊤} \end{array}$	$\begin{array}{ll} \text{For}\; & \Phi :\\ & \phi_{j}|\psi_{j}∼{𝒩}_{K}\;\left(0,\;\tau_{\Phi }^{2}\;\psi_{j}\;I_{K}\right),\\ & \psi_{j}∼\text{IG}\;\left(\frac{\nu_{0}}{2},\;\frac{\nu_{0}\psi_{0}}{2}\right)\\ \text{For}\; & \Lambda_{s}:\\ & \lambda_{js}|\psi_{js}∼{𝒩}_{J_{s}}\;\left(0,\;\tau_{\Lambda }^{2}\;\psi_{js}\;I_{J_{s}}\right),\\ & \psi_{js}∼\text{IG}\;\left(\frac{\nu_{0}}{2},\;\frac{\nu_{0}\psi_{0}}{2}\right)\\ \text{For}\; & \Psi_{s}:\\ & \psi_{ps}∼\text{IG}(a_{\psi },b_{\psi }) \end{array}$
Tetris	$\begin{array}{ll} y_{is} & =\Phi^{*}T_{s}f_{is}+\epsilon_{is},\\ T_{s} & =\text{diag}(t_{1s},\cdots \;,t_{Ks})\\ & \text{with}\;t_{ks}\in \{0,1\}\\ f_{is} & ∼\text{MVN}(0,I_{K})\\ \epsilon_{is} & ∼\text{MVN}(0,\Psi_{s})\\ \Psi_{s} & =\text{diag}(\psi_{1s},\psi_{2s},\ldots ,\psi_{Ps}) \end{array}$	$\begin{array}{ll} & \text{Marginal covariance:}\\ & \;\sum_{s}=\Phi^{*}T_{s}{\left(\Phi^{*}\right)}^{⊤}+\Psi_{s}\\ & \text{Common covariance:}\\ & \;\sum_{\Phi }=\Phi^{*}P{\left(\Phi^{*}\right)}^{⊤}\\ & \text{Study‐specific covariance:}\\ & \;\sum_{\Lambda_{s}}=\Phi^{*}R_{s}{\left(\Phi^{*}\right)}^{⊤}\\ & \text{where,}\;T_{s}=P+R_{s},\\ & \text{with}\;P=\text{diag}(1_{1},\cdots \;,1_{K^{*}}),\\ & \;1_{k}=\left\{\begin{array}{ll} 1\; & \text{if}\;t_{ks}=1\;\text{for all}\;s.\\ 0\; & \text{otherwise}. \end{array}\right. \end{array}$	$\begin{array}{ll} \text{For}\; & \Phi^{*}:\\ & \phi_{pk}^{*}|\omega_{pk},\theta ∼𝒩(0,\omega_{pk}^{-1}\theta_{k}^{-1})\\ & \omega_{pk}∼\text{Gam}(\kappa /2,\kappa /2)\\ & \theta_{k}=\prod_{l=1}^{k}\delta_{l},\;\delta_{1}∼\text{Gam}(a_{1},1),\\ & \delta_{l}∼\text{Gam}(a_{2},1),\;\text{for}\;l>1\\ \text{For}\; & T_{s}:\\ & 𝒯∼\text{Indian Buffet Process}(\alpha_{𝒯},\beta_{𝒯}),\\ & \text{where}\;s\text{th row of}\;𝒯\;\text{contains the}\\ & \text{diagonal entries of}\;T_{s}.\\ \text{For}\; & \Psi_{s}:\\ & \psi_{ps}∼\text{IG}(a_{\psi },b_{\psi }) \end{array}$

### 2.4 Identifiability Issues and Post‐Processing

A central challenge in factor models is rotational invariance, whereby the model remains unchanged under orthogonal transformations of the latent space. Specifically, if Φ denotes the factor loading matrix and F the matrix of factor scores, then for any orthogonal matrix Γ, the transformation $\Phi^{\prime }=\Phi \Gamma$ and $F^{\prime }=F\Gamma^{⊤}$ yields the same fitted values: $Y=F\Phi^{⊤}=F^{\prime }{\Phi^{\prime }}^{⊤}$. Two special cases of such transformations are column permutation (label switching) and sign flipping (multiplying a column by −1). In MCMC‐based approaches, posterior draws can differ by these transformations, leading to non‐identifiable or misleading posterior summaries (e.g., marginal means). Similarly, in EM‐based algorithms, multiple local optima can yield distinct but statistically equivalent solutions.

To address these issues, two broad strategies are commonly employed:

1. Impose parameters' constraints, such as assuming heteroscedastic factors or lower‐triangular loading matrices to break the rotational invariance.
2. Post‐processing the estimated loadings or posterior samples using rotation and alignment techniques, such as orthogonal Procrustes (OP) [63], etc.

Below, we briefly describe how each model in this tutorial (Stack FA, Ind FA, PFA, MOM‐SS, SUFA, BMSFA, Tetris) handles identifiability:

#### 2.4.1 Stack FA & Ind FA

Both Stack FA and Ind FA are estimated via a Gibbs sampling. To address identifiability *post hoc*, we adopt the Orthogonal Procrustes (OP) alignment [63], which aligns posterior samples to a reference loading matrix by minimizing the differences between the posterior draws and a reference loading matrix. This results in consistent orientation and factor labeling across MCMC draws.

Alternatively, one can use *Spectral Decomposition (SD)*. After estimating the covariance $\hat{\sum }$, SD decomposes it as $\hat{\sum }=UNU^{⊤}$, where U contains the eigenvectors and N the eigenvalues. Retaining the top $K^{*}$ eigenvectors and scaling by the square roots of their eigenvalues, the loadings matrix is reconstructed as $\hat{\Phi }=U_{K^{*}}\;N_{K^{*}}^{1/2}$. This is similar to standard PCA‐based factor analysis [64]. A third option is Varimax rotation [65], which seeks an orthogonal transformation that maximizes the variance of squared loadings within each factor. This approach enhances interpretability by encouraging sparsity and grouping high‐loading variables. More recent advancements addressing identifiability can be found in [66].

#### 2.4.2 PFA

Perturbed Factor Analysis (PFA) mitigates rotational ambiguity by assigning each latent factor its own variance (heteroscedastic factors). Specifically, post‐processing alignment is applied based on an extension of the Procrustes method, as described in Roy et al. [67] to provide uncertainty quantification (UQ) [67]. This approach yields consistent factor labeling across posterior draws.

#### 2.4.3 MOM‐SS

MOM‐SS model [34] applies a Varimax rotation during initialization to improve convergence and avoid spurious local maxima. Furthermore, the non‐local spike‐and‐slab prior [52] induces sparsity, shrinking many loadings toward zero, which helps reduce the space of equivalent solutions and mitigates label‐switching.

#### 2.4.4 SUFA

SUFA [35] addresses identifiability through a combination of design and post‐processing. First, it constrains the model via the inequality $\sum_{s=1}^{S}J_{s}\leq K$ and uses continuous priors on $A_{s}$ to avoid identifiability issues. Second, it applies a rotate‐and‐align procedure based on Varimax, as proposed in [68], to ensure a consistent orientation of the factors across MCMC draws.

#### 2.4.5 BMSFA

BMSFA [30] uses the OP method to resolve rotational invariance in the posterior samples, consistent with Stack FA and Ind FA.

#### 2.4.6 Tetris

Tetris [60] handles identifiability by selecting the posterior draw of 𝒯 (the factor activation matrix) that lies in the mode of the MCMC samples. Factor loadings are aligned across posterior draws by minimizing a mean‐squared error (MSE)‐like loss between each draw's implied covariance and the estimated marginal covariance. This effectively enforces consistent labeling and orientation across iterations.

#### 2.4.7 BLAST

BLAST [32] ensure identifiability, up to orthogonal rotations of the loadings, by imposing that the matrix obtained by concatenating Φ and $\Lambda_{s}^{\prime }$ has full rank.

#### 2.4.8 Practical Guidance and Applicability Across Models

In summary, all models discussed here face orthogonal non‐identifiability. However, the extent to which the user must intervene differs across methods. Stack FA, Ind FA, and BMSFA generally require an explicit post‐hoc step (typically OP, with SD as a covariance‐based alternative) to obtain interpretable factor‐level summaries. By contrast, PFA, MOM‐SS, SUFA, and Tetris incorporate constraints that largely address orientation within their estimation pipelines, with optional additional rotations (e.g., Varimax) to improve interpretability. Finally, independent of the chosen method, additional post‐processing such as factor reordering by explained variance or total absolute loadings [69] can be applied to highlight the most prominent factors.

#### 2.4.9 Software Note

To facilitate reproducible workflows, our software implements optional, method‐specific identifiability post‐processing for each model. In practice, users only need to call the function postprocess_*<method>* corresponding to the fitted model (e.g., postprocess_stackFA, postprocess_BMSFA, etc.), which applies the appropriate post‐process/rotation steps and returns consistently oriented loadings and factor scores. Because these procedures are method‐specific (e.g., OP/SD for Gibbs‐based FA and BMSFA, model‐tailored alignment for PFA and Tetris), the corresponding postprocess function should be used for the same method that generated the output, rather than transferred across models without modification. See the bmfaToolkits vignettes for illustrative examples and complete workflows.

### 2.5 Prior Specification

Bayesian factor analysis models require prior distributions for the factor loadings and idiosyncratic variances, together with hyperparameters that control shrinkage and regularization. These hyperparameters directly affect estimation stability, effective model complexity, and the extent to which the fitted loading matrix is sparse or dense.

Many methods reviewed in this tutorial rely on default priors, with hyperparameter values chosen to perform reasonably well across a range of applications. In practice, these defaults typically provide sufficient regularization to avoid extreme or unstable solutions while remaining flexible enough to capture meaningful latent structure. For applied users, they therefore offer a sensible starting point when prior information is limited.

Below, we summarize the prior specifications adopted by each method, highlight the practical role of key hyperparameters, and provide guidance on how users might adjust them to encourage stronger shrinkage or greater flexibility when needed. For detailed sensitivity analyses and additional guidance, we refer readers to the original methodological articles.

#### 2.5.1 Stack FA & Ind FA

Stack FA and Ind FA adopt the multiplicative gamma shrinkage prior [45] to impose increasing shrinkage on higher‐indexed factors, helping to stabilize estimation in high‐dimensional settings. Hyperparameters are set following the recommendations in [45, 50].

#### 2.5.2 PFA

PFA is typically implemented with default weakly informative priors for the factor loadings and for parameters for the number of factors. Variance components are assigned weakly informative priors as well, providing moderate regularization while allowing inference to be largely driven by the data.

#### 2.5.3 MOM‐SS

For the MOM‐SS spike‐and‐slab prior, the key hyperparameters are the variance parameters that control the spike and slab components. Following common practice, the spike variance is fixed at a small value to strongly shrink not important loadings toward zero. The slab variance is chosen to reflect practical relevance, leveraging the standard heuristic that (for standardized variables) squared loadings approximate the fraction of variance explained by a given factor. In particular, default values are selected so that loadings explaining less than 10% of the variance are treated as not important, while loadings exceeding this threshold are regarded as meaningfully different from zero. These defaults provide an interpretable and principled starting point when prior information is limited, and are designed to favor factors that explain a nontrivial portion of the observed variability.

#### 2.5.4 SUFA

SUFA uses the Dirichlet–Laplace shrinkage prior for the factor loadings, following the recommendations in Reference [53]. Default hyperparameter values are chosen to yield weakly informative priors, offering a practical starting point for applied analyses when strong prior knowledge is unavailable.

#### 2.5.5 BMSFA

BMSFA extends the multiplicative gamma shrinkage prior [45] to the multi‐study setting, using separate hyperparameters for shared and study‐specific loadings. Default hyperparameter values were calibrated via simulation studies across a range of scenarios, with the goal of providing robust estimation and good recovery of the covariance structure in practice. This empirical calibration offers a pragmatic starting point for applied multi‐study analyses.

#### 2.5.6 CAVI

The CAVI implementation also employs the multiplicative gamma shrinkage prior [45] and adopts the same default hyperparameters as BMSFA. While these defaults perform well in many settings, variational approximations can be sensitive to initialization and shrinkage strength; we therefore recommend basic sensitivity checks (e.g., varying shrinkage hyperparameters and/or using multiple random initializations) when applying CAVI in new data regimes.

#### 2.5.7 BLAST

BLAST adopts an empirical Bayes strategy to automatically select key prior hyperparameters, reducing the need for manual tuning. In particular, the prior variances controlling shrinkage for the common and study‐specific factor loadings are estimated directly from the data using moment‐based estimators. This approach calibrates the amount of shrinkage to the overall signal strength, allowing the model to adapt to different noise levels and degrees of heterogeneity across studies.

#### 2.5.8 Tetris

Tetris employs shrinkage priors on factor loadings and weakly informative priors for variance components, following the default recommendations in [30, 50]. These choices provide moderate regularization while allowing the data to primarily guide estimation, offering a practical starting point for applied analyses.

## 3 Simulation

### 3.1 Simulation Settings

We assess the performance of the nine previously described Bayesian integrative factor models in (1) recovering factor loadings (when true numbers of factors are known), and (2) estimating the number of latent factors (when numbers of factors are over‐specified) across multi‐study datasets with diverse latent structures.

The models are implemented as follows:

- StackFA, IndFA, and BMSFA using the MSFA R package (v0.86);
- PFA using the script “FBPFA‐PFA with fixed latent dim.R” from the GitHub repository https://github.com/royarkaprava/Perturbed‐factor‐model;
- MOM‐SS using the BFR.BE R package (v0.1.0);
- SUFA using the SUFA R package (v2.2.0);
- CAVI using the VIMSFA R package (v0.1.0);
- BLAST using the script from https://github.com/maurilorenzo/BLAST/tree/main;
- Tetris using the script from https://github.com/igrabski/tetris/tree/main.

Furthermore, we evaluate two versions of SUFA: SUFA_fixJs, which requires specifying the number of study‐specific factors, and SUFA, which infers this quantity automatically. Similarly, we evaluate two variants of Tetris: Tetris, the standard approach recommended by the author, and Tetris_fixT, which pre‐specifies the number of factors to improve scalability.

We consider six different simulation scenarios. The first three are based on the data‐generating processes from PFA, MOM‐SS, and SUFA, respectively, and are designed as small sample (per study) settings to reflect regimes where study‐specific structure may be difficult to recover. The fourth scenario is generated under the BMSFA mechanism and is constructed to have stronger study‐specific signals, allowing us to assess how increased study‐specific signal strength affects recovery. The fifth and sixth scenarios are more general and are designed to closely mimic the two case studies presented in Section 4; their data‐generating process is based on Tetris, which is more complex and involves more parameters than all the other methods.

For each scenario, we generated 50 collections of datasets. The data generation code is adapted from publicly available repositories of PFA, MOM‐SS, and BMSFA. In all scenarios, the data are centered (but not scaled) before model fitting, except for MOM‐SS, which incorporates study‐specific intercepts to account for uncentered data. For MCMC‐based methods, we run 10 000 iterations with a burn‐in of 8000. Post‐processing is carried out following the procedures described in Sections 2.4 and 4.2.4.

#### 3.1.1 Scenario 1: Based on PFA

In this scenario, data are generated from four studies (S=4), each with 100 samples ($N_{s}=100$), for a total of 400 observations, and the number of common factors K=4. Each sample includes 40 observed variables (P=40). The common factor loading matrix Φ is generated with 40% zero entries, and the remaining values are drawn from U(0.6,1) and assigned a negative sign with 0.5 probability. The residual covariance Ψ is set to be the same across all studies, with diagonal elements drawn from U(0,1). After generating $Y_{s}=F_{s}\Phi^{⊤}+E_{s}$, we apply a perturbation matrix $Q_{s}$ with perturbation level $\alpha_{Q}=0.01$ and compute $Y_{s}^{\text{perturbed}}=Q_{s}Y_{s}$.

The ground truth quantities used for evaluation include the common loadings Φ, the common covariance matrix $\sum_{\Phi }=\Phi \Phi^{⊤}+\Psi$, the study‐specific loadings and covariance matrices $\Lambda_{s}=(Q_{s}^{-1}-I_{P})\Phi$ and $\sum_{\Lambda_{s}}=\sum_{s}-\sum_{\Phi }$, and the marginal covariances $\sum_{s}=Q_{s}\sum_{\Phi }Q_{s}^{⊤}$. Note that if we derive the study‐specific loadings as above, the first study has $\Lambda_{1}=0$ since $Q_{1}=I_{P}$. However, we can use $\Lambda_{1}=\Phi$ and $\sum_{\Lambda_{1}}=\sum_{\Phi }$, since we know that other studies are inherently perturbed from the first study, that is, the signals of the first study is actualy the common signal.

Each method is fit with the true number of common factors K=4, with the excepstion of IndFA, which does not include the common factors and thus we set the number of study‐specific factors $J_{s}$ to 4 for each study, since the true $\Lambda_{s}=(Q_{s}^{-1}-I_{P})\Phi$ are loadings of dimension P×K, that is, $J_{s}=K$. For other models that require $J_{s}$, we set $J_{s}=4$ for SUFA_fixJs, BMSFA, CAVI and BLAST. For Tetris_fixT, we predefine the matrix for factor structure 𝒯 to include 4 common factors and 4 study‐specific factors for each study. Tetris also has a version that does not require pre‐specification of K and $J_{s}$.

#### 3.1.2 Scenario 2: Based on MOM‐SS

In this scenario, the data are again generated from four studies (S=4), each consisting of 100 samples, for a total of 400 observations. Each sample includes 40 observed variables (P=40), and the data‐generating process incorporates common factors (K=4), study‐specific intercepts ($\alpha_{s}$), two observed covariates (Q=2), and noise terms drawn from a multivariate normal distribution with study‐specific idiosyncratic covariance matrices. The common loading matrix Φ is constructed to be sparse, with 40% of entries set to zero. The remaining entries are drawn uniformly from the interval [0.6,1] and randomly assigned negative signs with probability 0.5.

The quantities used for evaluation in this scenario include the common loading matrix Φ, the common covariance matrix $\sum_{\Phi }=\Phi \Phi^{⊤}$, and the total covariances in each study, $\sum_{s}=\Phi \Phi^{⊤}+\Psi_{s}$. Since MOM‐SS does not include study‐specific factor loadings in its generative process, there are no true $\Lambda_{s}$ or $\sum_{\Lambda_{s}}$ in this setting.

For model fitting, we use the true values K=4 and $J_{s}=1$ wherever the method requires them. In the case of IndFA, we set $J_{s}=5$ for each study. For CAVI we set $J_{s}=2$ since it does not accept $J_{s}=1$ when running the model.

#### 3.1.3 Scenario 3: Based on SUFA

In this scenario, data are generated from four studies (S=4), each containing 100 samples, for a total of 400 observations. Each sample includes 40 observed variables (P=40), generated from a multivariate normal distribution with covariance structure $\sum_{s}=\Phi \Phi^{⊤}+\Phi A_{s}A_{s}^{⊤}\Phi^{⊤}+\Psi$. The common loading matrix Φ is generated with K=4 factors and follows the same construction as in the previous scenarios, with 40% sparsity and nonzero entries drawn from a uniform distribution on [0.6,1], with random sign flipping. The study‐specific factor structure is introduced via the matrices $A_{s}$, which are drawn from a multivariate normal distribution centered at zero with standard deviation 0.4.

The true quantities used for evaluation are the common loadings Φ, the common covariance $\sum_{\Phi }=\Phi \Phi^{⊤}+\Psi$, the study‐specific loadings $\Lambda_{s}=\Phi A_{s}$, the study‐specific covariances $\sum_{\Lambda_{s}}=\Phi A_{s}A_{s}^{⊤}\Phi^{⊤}$, and the marginal covariances $\sum_{s}$.

For model fitting, we use the true values K=4 and $J_{s}=1$ wherever the method requires them. In the case of IndFA, we set $J_{s}=5$ for each study. For CAVI, we set $J_{s}=2$ for the same reason as in Scenario 2.

#### 3.1.4 Scenario 4: BMSFA Data‐Generating Mechanism With Strong Study‐Specific Signals

This scenario is designed to assess whether the methods can reliably recover study‐specific structure when (i) per‐study sample sizes are larger and (ii) study‐specific signal is stronger and less sparse. In particular, we generate data with more study‐specific factors than shared factors, denser loading matrices, and larger within‐study sample sizes. We generate data for four studies (S=4), each with $N_{s}=200$ samples and P=50 observed variables. The data are generated with K=2 common factors and $J_{s}=5$ study‐specific factors per study. Both the shared loadings Φ and study‐specific loadings $\Lambda_{s}$ are taken to be relatively dense, with 25% of entries set to zero. Nonzero entries are drawn independently from Unif(0.6,1). For each study, observations $Y_{s}$ are generated with covariance structure $\sum_{s}=\Phi \Phi^{⊤}+\Lambda_{s}\Lambda_{s}^{⊤}+\Psi_{s}$, where $\Psi_{s}$ is diagonal with entries drawn from Unif(0,1).

The true quantities used for evaluation include the common loadings Φ, the study‐specific loadings $\Lambda_{s}$, the common covariance matrix $\sum_{\Phi }=\Phi \Phi^{⊤}$, the study‐specific covariance matrices $\sum_{\Lambda_{s}}=\Lambda_{s}\Lambda_{s}^{⊤}$, and the marginal covariances $\sum_{s}=\Phi \Phi^{⊤}+\Lambda_{s}\Lambda_{s}^{⊤}+\Psi_{s}$.

For model fitting, we set K=2 for all methods. For SUFA_fixJs, BMSFA, CAVI, and BLAST, we set $J_{s}=5$ for each study. For IndFA, we set $J_{s}=7$ to provide flexibility for modeling potentially shared factors. We set K=3 for PFA because it does not allow K≤2. Tetris_fixT is fit using the same structure 𝒯 as used in data generation, ensuring optimal alignment with the truth.

#### 3.1.5 Scenario 5: Mimic Nutrition Data and Based on Tetris

In this scenario, the data are designed to closely mimic the real case nutrition dataset analyzed in Section 4. Data are generated from twelve studies (S=12), with varying sample sizes per study: $N_{s}=(1362,217,417,1012,2241,205,2403,3775,1790,761,373,465)$, resulting in a total sample size of 16 021. Each study includes 42 observed variables (P=42) and 12 covariates X.

For each individual, the observed response vector $y_{is}$ is generated from a multivariate normal distribution with covariance $\sum_{s}=\Phi^{*}T_{s}{\left(\Phi^{*}\right)}^{⊤}+\Psi_{s}$, following the Tetris model. We also added the covariates and random residuals. The combinatorial loading matrix $\Phi^{*}$ is generated as in the previous scenarios, with sparsity and uniformly distributed non‐zero entries. The latent structure encoded in $T_{s}$ assigns K=4 common factors, $J_{s}=1$ study‐specific factor for each study, and 7 partially shared factors distributed across studies.

The ground truth includes the common loadings Φ, the study‐specific loadings $\Lambda_{s}$, the common covariance $\sum_{\Phi }$, the study‐specific covariances $\sum_{\Lambda_{s}}$, and the marginal covariances $\sum_{s}$, as summarized in Table 1.

When fitting the models, we specify K=4 for all methods. For SUFA_fixJs, BMSFA and BLAST, we set $J_{s}=1$ for each study, acknowledging that this may slightly violate the identifiability conditions for SUFA. For IndFA, we specify $J_{s}=5$ to provide flexibility for modeling potentially shared factors. For CAVI we set K=4 and $J_{s}=2$. Tetris_fixT is fit using the exact structure 𝒯 employed during data generation, ensuring optimal alignment with the truth.

#### 3.1.6 Scenario 6: Mimic Gene Expression Data and Based on Tetris

In this final scenario, the data are constructed to closely mimic the real case study of gene expression used in Section 4, that is, curatedOvarianData [70]. The simulation includes four studies (S=4), with study‐specific sample sizes given by $N_{s}=(157,195,285,117)$. Each sample contains high‐dimensional P=1060 gene expression variables. The data are generated under the Tetris model, incorporating K=15 common factors, $J_{s}=2$ study‐specific factors for each study, and 3 partially shared factors.

The loading matrix $\Phi^{*}$ is generated as in previous scenarios, but with increased sparsity: 80% of its entries are set to zero. The non‐zero elements are drawn from a uniform distribution over [0.6,1] and are randomly signed. The covariance matrices $\sum_{s}$ are constructed according to the Tetris model: $\sum_{s}=\Phi^{*}T_{s}{\left(\Phi^{*}\right)}^{⊤}+\Psi_{s}$. The true quantities for evaluation are the common loadings Φ, study‐specific loadings $\Lambda_{s}$, common covariance $\sum_{\Phi }$, study‐specific covariances $\sum_{\Lambda_{s}}$, and marginal covariances $\sum_{s}$, as detailed in Table 1.

For model fitting, we set K=15 for all methods, and $J_{s}=2$ for SUFA_fixJs, BMSFA, CAVI, and BLAST. For IndFA, we increase flexibility by specifying $J_{s}=17$ for each study, to account for both study‐specific and potentially shared variation. Tetris_fixT is applied using the exact 𝒯 matrix of data generation. Due to high computational demands, SUFA and SUFA_fixJs are only repeated 30 times in this scenario.

#### 3.1.7 Over‐specifying the Number of Factors

To assess the ability of each method to recover the correct number of common (K) and study‐specific ($J_{s}$) factors, we repeat the simulations from all six scenarios, this time over‐specifying the number of factors when fitting the models. Tetris, which internally determines its latent structure, is evaluated using the same results as in the correctly specified setting. SUFA_fixJs and Tetris_fixT are not run due to their reliance on fixed, known factor configurations.

For Scenario 1 (true K=4 and true $J_{s}=4$), we set K=6 for StackFA, PFA, MOM‐SS, SUFA, BMSFA, and CAVI, and set $J_{s}=5$ for BMSFA and CAVI. for IndFA, we set $J_{s}=6$ in each study. For BLAST, we set $k_{\max}$ (the total number of factors, that is, $K+\sum_{s}^{S}J_{s}$) to 25.

Using the same principle, for Scenarios 2 and 3, we set K=6, and $J_{s}=2$ in BMSFA and CAVI, and $J_{s}=6$ in IndFA, for all studies (s=1,…,4). In BLAST, we set $k_{max}=8$.

For Scenario 4, we set K=6 for StackFA and $J_{s}=10$ for IndFA. For MOM‐SS and SUFA, we set K=4. For BMSFA and CAVI, we set K=4 and $J_{s}=8$. For BLAST, we set $k_{\max}=25$.

For Scenario 5, we specify K=6 for StackFA, PFA, MOM‐SS, SUFA, BMSFA, and CAVI, $J_{s}=2$ for BMSFA and CAVI, and $J_{s}=6$ for IndFA across all twelve studies. For BLAST, we set $k_{\max}=20$.

For Scenario 6, we set K=20 in StackFA, MOM‐SS, SUFA, BMSFA, and CAVI, $J_{s}=4$ for BMSFA and CAVI, and $J_{s}=20$ for IndFA, for s=1,…,4. For BLAST, we set $k_{\max}=20$.

For models that estimate the number of factors internally, namely MOM‐SS, SUFA, BLAST, and Tetris, we extract the inferred K and $J_{s}$ directly from the dimensions of their estimated loading matrices. For PFA, which uses an adaptive truncation strategy within the MCMC procedure, we estimate K by computing the mode of the number of active columns across posterior samples. In contrast, StackFA, IndFA, BMSFA, and CAVI do not estimate the number of factors directly. Instead, we apply eigenvalue decomposition (EVD) to their estimated covariance matrices and determine the number of factors as those explaining more than 5% of the total variance.

### 3.2 Simulation Results: Factor Loadings and Computational Efficiency

We evaluate the performance of each model in recovering the common loadings Φ, study‐specific loadings $\Lambda_{s}$, common covariances $\sum_{\Phi }$, study‐specific covariances $\sum_{\Lambda_{s}}$, and marginal covariances $\sum_{s}$ by comparing them to the ground truth. Two metrics are used: The RV coefficient [71] and the Frobenius norm (FN) [72] (see Supporting Information for full FN definitions and results).

The RV coefficient, implemented via the RV function in the R package MatrixCorrelation [73], measures matrix similarity and is defined as:

(19) $$\text{RV}(X,Y)=\begin{array}{c} \frac{\text{trace}\{XX^{⊤}YY^{⊤}\}}{\sqrt{\left(\text{trace}\{XX^{⊤}XX^{⊤}\})\times (\text{trace}\{YY^{⊤}YY^{⊤}\}\right)}} \end{array}$$

It ranges from 0 (no similarity) to 1 (perfect similarity). The Frobenius norm quantifies the element‐wise difference between two matrices; values closer to 0 indicate better estimation accuracy. For study‐specific quantities, RV and FN scores are averaged across studies.

Computational performance is assessed by recording runtime (in seconds) and peak memory usage (in MiB).

**FIGURE 2:**
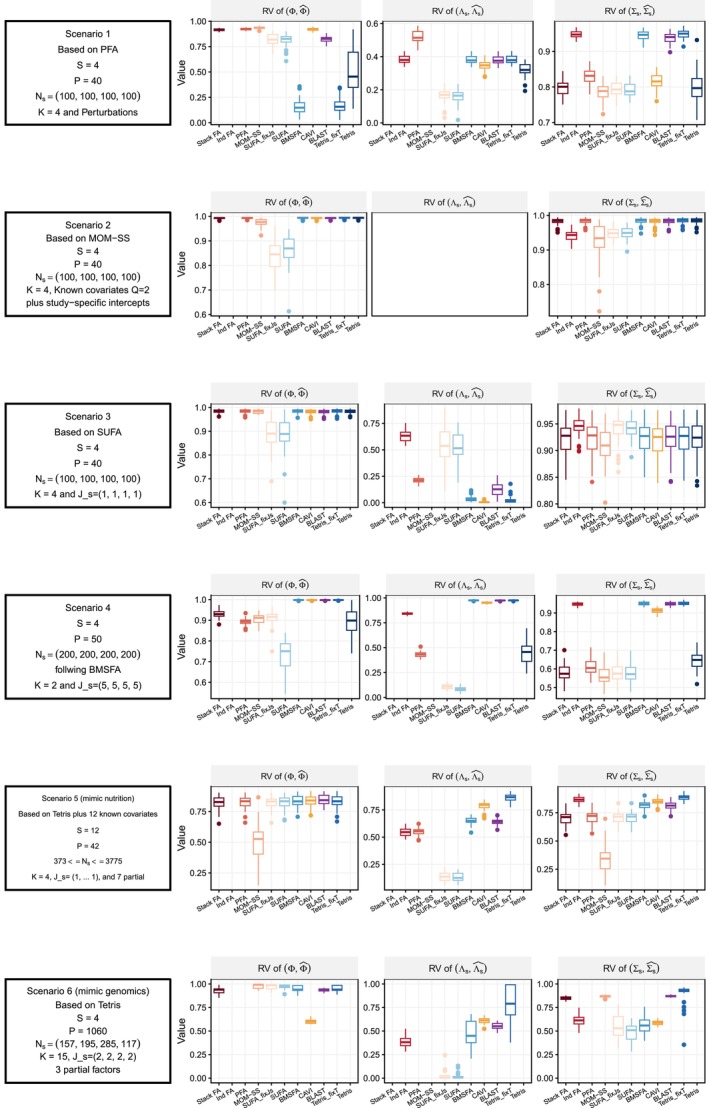
Measuring accuracy of different Bayesian integrative factor models in estimating factor loadings and marginal covariance matrices, across five simulation scenarios with correctly specified numbers of factors. The first column lists out the settings of a scenario, the second column shows the RV coefficient between estimated and true common loadings (Φ), the third column shows the RV or study‐specific loadings ($\Lambda_{s}$), and the third column shows the RV for the marginal covariance matrices ($\sum_{s}$). In Scenarios 1 and 2, the study‐specific loadings $\Lambda_{s}$ are not part of the generative model (PFA and MOM‐SS, respectively), so the corresponding panels are blank. StackFA and IndFA estimate only common or study‐specific loadings, respectively, and thus may have missing values in some panels. PFA fails to run in Scenario 5 due to memory and time constraints. Tetris runs for over 24 h in Scenarios 4 and 5. Thus, we do not report results for those models in those scenarios.

**FIGURE 3:**
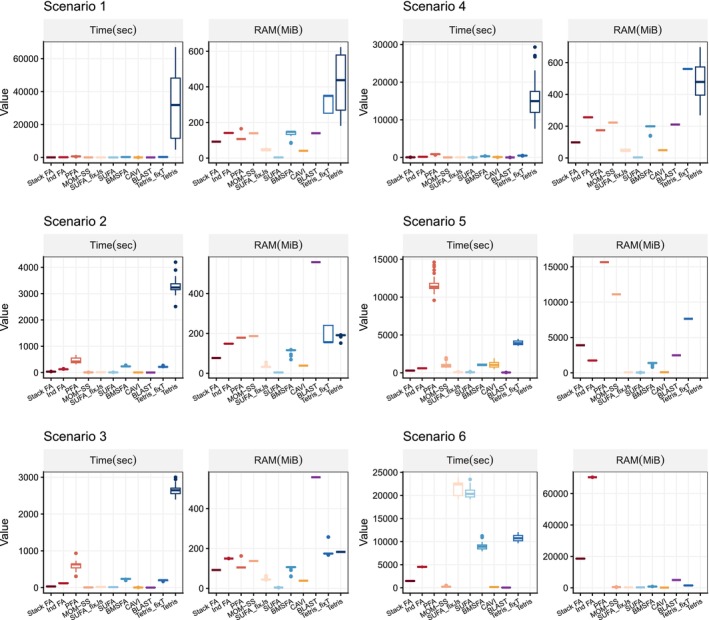
Computational efficiency of Bayesian integrative factor models, evaluated under correctly specified numbers of factors across the five simulation scenarios. The runtime (in seconds) and peak memory usage (in MiB) are recorded for each model.

Figure 2 summarizes the accuracy of each method across the scenarios considered, whereas Figure 3 reports the corresponding computational time (in seconds) and peak memory usage (in MiB) for each method in each scenario. Both figures report results under the setting where the true number of factors is assumed to be known. In Scenario 1 (PFA‐generated data), most models accurately recover the common loadings and marginal covariances, although BMSFA and Tetris perform less well and Tetris takes substantially longer due to the difficulty in identifying small perturbations.

In Scenario 2 (MOM‐SS), all models perform well despite the presence of known covariates, since these effects are removed during data centering or captured in the study‐specific effects. In Scenario 2, Stack FA, MOM‐SS, SUFA, CAVI, and BLAST achieve the shortest runtimes, whereas SUFA exhibits the lowest peak memory usage. Scenario 2 does not include the generation of study‐specific loadings $\Lambda_{s}$. As a result, $\Lambda_{s}$ is not used for precision comparisons in this setting; therefore, the corresponding panels for $\Lambda_{s}$ in Figure 2 are blank.

Scenario 3, generated from SUFA, features study‐specific loadings constructed as $\Lambda_{s}=\Phi A_{s}$ with small perturbations. As a result, many models recover Φ and $\sum_{s}$ well, but fail to accurately estimate $\Lambda_{s}$; in particular, PFA, BMSFA, CAVI, BLAST and Tetris are less successful, with Tetris failing to detect any study‐specific variation due to its weak signal. Scenario 3 exhibits a similar pattern to Scenario 2 in terms of computational time, with Stack FA, MOM‐SS, and SUFA remaining the fastest methods.

In Scenario 4, where the data‐generating mechanism is constructed to exhibit a strong study‐specific signal, BMSFA, CAVI, BLAST, and Tetris_fixT provide the best overall performance, achieving the highest accuracy while remaining computationally efficient. SUFA performs well for estimating Φ when the number of study‐specific factors is correctly specified, but it underperforms in recovering the study‐specific loadings $\Lambda_{s}$. StackFA, MOM‐SS, and IndFA perform well, but their structural assumptions limit them to recovering only shared components (StackFA) or only study‐specific components (IndFA), with MOM‐SS offering an intermediate compromise. In contrast, PFA and Tetris exhibit weaker accuracy in this scenario, consistent with their reduced ability to accommodate strong study‐specific effects without additional structure. From a computational perspective, for this moderately sized setting, most methods complete within one hour, whereas Tetris can require up to approximately eight hours.

In Scenario 5, which mimics the real nutrition data structure ($N_{s}\gg P$), Tetris_fixT produces the most accurate estimates across all quantities due to perfect alignment with the generative model. BMSFA also performs well, but cannot account for partially shared factors. PFA achieves good accuracy but is computationally expensive, especially in memory usage. MOM‐SS converges quickly, but struggles with precision when N is large, probably due to premature termination of the EM algorithm or convergence to a local maximum. SUFA underestimates study‐specific loadings due to its model assumptions. CAVI and BLAST show their benefit in dealing with large sample size data—they outperform in time and memory used, while accuracy in estimating loadngs are preserved, even when partially shared factors are overlooked by these two methods.

In Scenario 6, which mirrors high‐dimensional genomic data ($P\gg N_{s}$), most models estimate the common loadings accurately, even though the loadings are designed to be sparser in this scenario. Tetris_fixT again performs best due to its alignment with the underlying structure. SUFA remains comparable in accuracy to Scenario 5 but requires significantly more computational resources, as its complexity scales with P. PFA fails to complete within 24 h and shows sensitivity to the dimensionality of the data. MOM‐SS, CAVI, and BLAST exhibit great advantages in their efficiency when dealing with data with large P.

Overall, model performance is scenario‐dependent. StackFA and IndFA are limited by their structural assumptions, recovering only common or study‐specific components but not both. These two methods, used as benchmarks, also require a lot of memory. PFA is accurate but slow, particularly with large P, due to the estimation of the $Q_{s}$ matrix of size P×P for each study. MOM‐SS runs quickly using an EM algorithm, but loses accuracy in complex scenarios, especially when the number of studies increases, and tends to overestimate loadings. SUFA is efficient when P is small, even for large $N_{s}$, and performs consistently across scenarios, although its speed advantage diminishes when P is large, and there is a moderate variance of the accuracy across scenarios. SUFA is also limited by the constraint $\sum_{s=1}^{S}J_{s}\leq K$; when the study‐specific numbers of factors $J_{s}$ are large, this restriction can obscure accurate estimation of the study‐specific loading structure. BMSFA is robust across scenarios but incurs longer runtimes due to Gibbs sampling. CAVI and BLAST are fast implementations of BMSFA, which are fast and accurate in all scenarios. BLAST requires slightly more memory use in some situations (Scenarios 2 and 3). Tetris, while highly accurate, does not scale well to high‐dimensional data. Its simplified variant, Tetris_fixT, slightly reduces time and memory requirements while maintaining high estimation accuracy.

Models using MCMC algorithms, such as BMSFA, PFA, and Tetris, generally incur higher computational costs. MOM‐SS is the fastest method overall due to its EM‐based estimation, while SUFA remains efficient in scenarios with small to moderate P. Tetris shows the highest computational cost in Scenarios 5 and 6, exceeding 24 h of runtime; PFA also fails to complete Scenario 6 within the time limit. Thus, results for Tetris in Scenarios 5 and 6 and for PFA in Scenario 6 are not available. Tetris_fixT provides a faster and more memory‐efficient alternative to Tetris while maintaining competitive accuracy. Tetris estimates numbers of factors all by itself, therefore loses accuracy and efficiency.

### 3.3 Simulation Results: Estimating the Number of Factors

Table 2 summarizes the results. No method accurately recovers the correct number of factors across all scenarios. EVD‐based approaches (used for StackFA, IndFA, BMSFA, and CAVI) perform well in Scenarios 1–4. However, in Scenario 6, these methods tend to underestimate both K and $J_{s}$, likely due to the high sparsity of the true loading matrix. PFA and MOM‐SS consistently overestimate K, indicating overly permissive inclusion thresholds. SUFA also tends to overestimate K, whereas its estimates of $J_{s}$ remain stable across studies and scenarios, due to its identifiability constraints, reflecting the model's identifiability constraint $\sum_{s=1}^{S}J_{s}\leq K$. BLAST accurately recovers both the shared dimension K and the study‐specific dimensions $J_{s}$ in Scenarios 2, 3, 4, and 6, but performs less well in Scenario 1, where the data are generated under PFA. Notably, BLAST's estimates of $J_{s}$ in Scenario 1 remain consistent with the structure induced by the PFA data‐generating mechanism, and thus still reflect the underlying characteristics of that model.

**TABLE 2:** Results for estimated K and $J_{s}$ when the number of factors is over‐specified.

Model	Estimated K	Estimated $J_{s}$
Scenario 1: True $K=4,J_{s}=(4,4,4,4)$		
Stack FA	4.10 (0.30)	—
Ind FA	—	4.00 (0.00), 4.00 (0.00), 4.00 (0.00), 4.00 (0.00)
PFA	6.00 (0.00)	—
MOM‐SS	6.00 (0.00)	—
SUFA	6.00 (0.00)	1.00 (0.00), 1.00 (0.00), 1.00 (0.00), 1.00 (0.00)
BMSFA	5.50 (0.54)	4.00 (0.00), 4.00 (0.00), 4.00 (0.00), 4.00 (0.00)
CAVI	4.00 (0.00)	2.94 (0.51), 3.92 (0.34), 3.80 (0.49), 3.86 (0.40)
BLAST	3.58 (0.50)	1.00 (0.00), 21.42 (0.50), 21.42 (0.50), 21.42 (0.50)
Tetris	1.32 (1.34)	5.12 (2.44), 11.32 (5.00), 11.58 (5.18), 10.80 (4.49)
Scenario 2: True $K=4,J_{s}=(0,0,0,0)$		
Stack FA	4.00 (0.00)	—
Ind FA	—	4.00 (0.00), 4.00 (0.00), 4.00 (0.00), 4.00 (0.00)
PFA	6.00 (0.00)	—
MOM‐SS	6.00 (0.00)	—
SUFA	5.98 (0.14)	1.00 (0.00), 1.00 (0.00), 1.00 (0.00), 1.00 (0.00)
BMSFA	4.00 (0.00)	2.00 (0.00), 2.00 (0.00), 2.00 (0.00), 2.00 (0.00)
CAVI	4.00 (0.00)	1.94 (0.24), 1.88 (0.33), 1.92 (0.27), 1.96 (0.20)
BLAST	4.00 (0.00)	1.00 (0.00), 1.00 (0.00), 1.00 (0.00), 1.02 (0.14)
Tetris	4.98 (0.25)	0.22 (0.42), 0.18 (0.39), 0.26 (0.49), 0.20 (0.40)
Scenario 3: True $K=4,J_{s}=(1,1,1,1)$		
Stack FA	4.00 (0.00)	—
Ind FA	—	4.00 (0.00), 4.00 (0.00), 4.00 (0.00), 4.00 (0.00)
PFA	6.00 (0.00)	—
MOM‐SS	6.00 (0.00)	—
SUFA	5.98 (0.14)	1.00 (0.00), 1.00 (0.00), 1.00 (0.00), 1.00 (0.00)
BMSFA	4.00 (0.00)	2.00 (0.00), 2.00 (0.00), 2.00 (0.00), 2.00 (0.00)
CAVI	4.00 (0.00)	2.00 (0.00), 2.00 (0.00), 2.00 (0.00), 2.00 (0.00)
BLAST	4.00 (0.00)	1.00 (0.00), 1.00 (0.00), 1.00 (0.00), 1.00 (0.00)
Tetris	4.00 (0.00)	0.00 (0.00), 0.00 (0.00), 0.00 (0.00), 0.00 (0.00)
Scenario 4: True $K=2,J_{s}=(5,5,5,5)$		
Stack FA	6.00 (0.00)	—
Ind FA	—	6.98 (0.14), 6.98 (0.14), 6.96 (0.20), 6.98 (0.14)
PFA	4.00 (0.00)	—
MOM‐SS	4.00 (0.00)	—
SUFA	4.00 (0.00)	1.00 (0.00), 1.00 (0.00), 1.00 (0.00), 1.00 (0.00)
BMSFA	1.72 (0.50)	5.34 (0.52), 5.32 (0.47), 5.34 (0.52), 5.34 (0.52)
CAVI	4.00 (0.00)	4.82 (0.39), 4.90 (0.30), 4.88 (0.33), 4.94 (0.24)
BLAST	2.00 (0.00)	5.36 (2.55), 5.36 (2.55), 6.44 (4.93), 5.36 (2.55)
Tetris	5.74 (1.69)	4.58 (2.60), 4.96 (2.70), 4.44 (2.69), 4.80 (2.66)
Scenario 5: True $K=4,J_{s}=(1,1,1,1,1,1,1,1,1,1,1,1)$		
Stack FA	6.00 (0.00)	—
Ind FA	—	6.00 (0.00), 6.00 (0.00), 6.00 (0.00), 6.00 (0.00), 6.00 (0.00), 6.00 (0.00), 6.00 (0.00), 6.00 (0.00), 6.00 (0.00), 6.00 (0.00), 6.00 (0.00), 6.00 (0.00)
PFA	6.00 (0.00)	—
MOM‐SS	6.00 (0.00)	—
SUFA	6.00 (0.00)	1.00 (0.00), 1.00 (0.00), 1.00 (0.00), 1.00 (0.00), 1.00 (0.00), 1.00 (0.00), 1.00 (0.00), 1.00 (0.00), 1.00 (0.00), 1.00 (0.00), 1.00 (0.00), 1.00 (0.00)
BMSFA	6.00 (0.00)	2.00 (0.00), 2.00 (0.00), 2.00 (0.00), 2.00 (0.00), 2.00 (0.00), 2.00 (0.00), 2.00 (0.00), 2.00 (0.00), 2.00 (0.00), 2.00 (0.00), 2.00 (0.00), 2.00 (0.00)
CAVI	6.00 (0.00)	2.00 (0.00), 2.00 (0.00), 2.00 (0.00), 2.00 (0.00), 2.00 (0.00), 2.00 (0.00), 2.00 (0.00), 2.00 (0.00), 2.00 (0.00), 2.00 (0.00), 2.00 (0.00), 2.00 (0.00)
BLAST	15.98 (0.43)	4.02 (0.43), 4.02 (0.43), 4.02 (0.43), 4.02 (0.43), 4.02 (0.43), 4.02 (0.43), 4.02 (0.43), 4.02 (0.43), 4.02 (0.43), 4.02 (0.43), 4.02 (0.43), 4.02 (0.43)
Scenario 6: True $K=15,J_{s}=(2,2,2,2)$		
Stack FA	10.90 (1.04)	—
Ind FA		5.06 (2.24), 7.10 (1.58), 10.84 (0.91), 2.34 (1.57)
MOM‐SS	20.00 (0.00)	—
SUFA	19.00 (0.00)	4.00 (0.00), 4.00 (0.00), 4.00 (0.00), 4.00 (0.00)
BMSFA	11.76 (0.85)	2.00 (0.00), 2.00 (0.00), 1.70 (0.46), 1.94 (0.24)
CAVI	5.50 (0.63)	3.94 (0.25), 3.94 (0.25), 4.00 (0.00), 3.94 (0.25)
BLAST	15.00 (0.00)	4.00 (0.00), 5.00 (0.00), 3.00 (0.00), 5.00 (0.00)

*Note:* Values are presented with mean(SD) over 50 datasets; “—” indicates the method does not defined with either common or study‐specific factors; CAVI runs into errors in 34 out of 50 replicates in Scenario 6, thus results are reported with the remaining 16 replicates only.

Tetris does not accurately recover K and $J_{s}$ in Scenarios 1–3. Specifically, in Scenario 1, it fails to detect the small perturbation effects encoded in the data, leading to an underestimation of K and an overestimation of $J_{s}$, which also contributes to its long runtime. In Scenario 2, Tetris overestimates K but gives the closest estimates for $J_{s}$. In Scenario 3, Tetris does not recover any study‐specific factors, likely due to the small magnitude of $\Lambda_{s}$ generated via SUFA.

## 4 Case Study Demonstration

### 4.1 Package Installation and Environment Setup

This tutorial is accompanied by an open‐source R package bmfaToolkits, and a reproducible code repository, designed to guide researchers through the implementation and comparison of the Bayesian multi‐study factor models introduced in this paper. bmfaToolkits is available on GitHub at https://github.com/Mavis‐Liang/bmfaToolkits usage documentation is available at https://mavis‐liang.github.io/bmfaToolkits/articles/toy_workflows.html.

Our repository requires R version 4.4.0 (or later, 4.5.1 is recommended) to support the installation of Matrix package version 1.7.

#### 4.1.1 Installing the bmfaToolkits Package

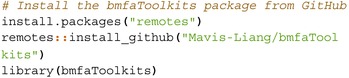

PFA, BLAST, and Tetris are included in the bmfaToolkits package as R scripts and can be invoked directly. The remaining models can be installed using the following code.

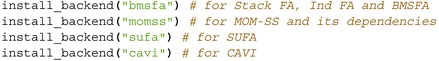

However, we recommend installing MSFA and BFR.BE, SUFA, and CAVI packages manually to facilitate troubleshooting. Installation instructions for each model are provided below.

#### 4.1.2 Stack FA, Ind FA and BMSFA

The Stack FA, Ind FA, and BMSFA models are implemented in the MSFA package, which is available on GitHub and can be installed using the remotes package:

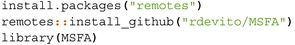

The function sp_fa() is used to fit both the Stack FA model (using pooled data) and the Ind FA model (fitted separately to each study). The BMSFA model is fitted using sp_msfa().

#### 4.1.3 MOM‐SS

The MOM‐SS model is implemented in the BFR.BE package, which is available on GitHub. The package depends on sparseMatrixStats and the mombf package, both of which must be installed prior to installation. The following code installs the required dependencies and loads the package:

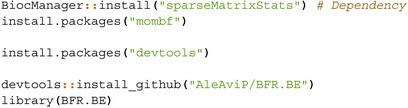

#### 4.1.4 SUFA

The SUFA package can be installed from GitHub. On Linux systems, installation requires additional system dependencies, such as PROJ, sqlite3, and GDAL, to be available in the system PATH. On Windows, additional updates, particularly for the terra package, may be necessary. During installation, building the package vignettes can be skipped to reduce installation time, as they involve computationally intensive datasets.

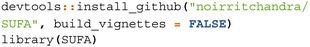

#### 4.1.5 CAVI

CAVI can be installed from the GitHub repository.

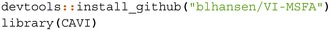

##### 4.1.5.1 Other Utility Packages

The following R packages are also used throughout the tutorial for data manipulation, visualization, and matrix operations:

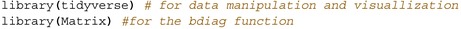

### 4.2 Case 1: Nutrition Data Analysis

#### 4.2.1 The Data

For the first case study, we analyze data from the Hispanic Community Health Study/Study of Latinos (HCHS/SOL), a large, multi‐site cohort study investigating health and dietary habits among Hispanic/Latino adults in the United States. The data comprises 24‐hour dietary recall data collected between 2008 and 2011 from 16 415 participants across four sites (Bronx, Chicago, Miami, and San Diego), and six ethnic backgrounds (Mexican, Puerto Rican, Cuban, Dominican, Central American, and South American) [74].

We focus our analysis on 42 key nutrients selected to best represent the overall diet habits, with an emphasis on those related to cardiovascular health. Following the approach of [75], we perform the multi‐study techniques by treating individuals from different ethnic backgrounds as separate studies (S=6).

This case study has two primary objectives:

i. to estimate nutritional patterns that are both shared and specific across the six ethnic groups, and
ii. to assess the out‐of‐sample predictive performance of the various integrative factor analysis methods.

The processed dataset is structured as a list of length S=6, where each element is a data frame of dimension $N_{s}\times P$, with P=42 nutrients and $N_{s}=(1364,\;1517,\;2210,\;5184,\;2478,\;959)$ representing the number of individuals in each ethnic group.

#### 4.2.2 Pre‐Processing

We begin by excluding individuals with missing nutrient intake values, missing background information, or extreme total energy intake values, resulting in a final sample size of N=10460. Subjects reporting negative nutrient intakes are also removed. This results in a final sample of N=10460 individuals.

Nutrient intake values are transformed using a log transformation, log(value+0.01), to improve adherence to normality assumptions.

For each study, the nutrient data are mean‐centered column‐wise, except for MOM‐SS, where mean‐centering is unnecessary because the model explicitly estimates random intercepts.

#### 4.2.3 Model Fitting

We fit each method using K=6 common factors and $J_{s}=2$ ethnic background specific factors, which are reasonable upper bounds based on previous analysis [75]. The following code demonstrates how each model is estimated using default parameters discussed in Section 2:

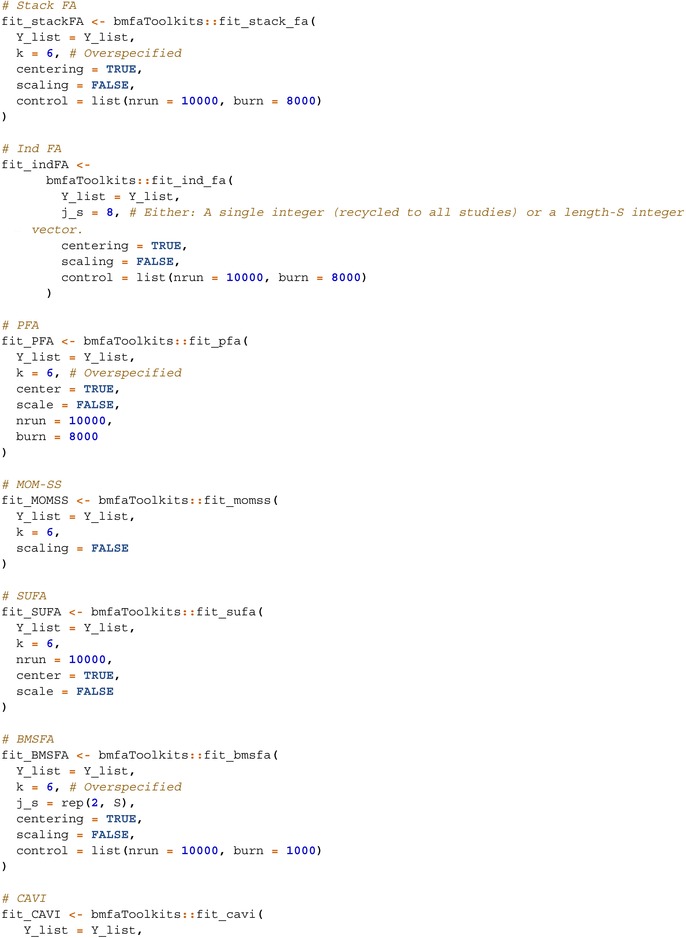

Unlike the other methods, fitting Tetris involves a three‐step procedure. Posterior samples of factor loadings are not directly comparable between different realizations of the factor sharing matrix 𝒯, which complicates direct inference. As recommended by the authors, we first use the tetris function to sample from the posterior distribution of all model parameters, including 𝒯. We then apply the choose.A function to obtain a point estimate of 𝒯. Finally, we rerun the sampler with 𝒯 fixed to this estimate to generate posterior samples for factor loadings.

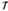

In practice, model fitting should be conducted in a high‐performance computing environment, as some methods are computationally demanding. In our experiments, PFA required more than 10 h to complete, while Tetris took approximately 4 days to run on a high‐performance computing cluster at Brown University. This cluster, managed by the Center for Computation and Visualization, consists of 388 compute nodes with a total of 20 176 CPU cores. In contrast, all other models were completed in under 30 min.

#### 4.2.4 Post‐processing

This section describes the post‐processing steps used to estimate the number of factors and obtain point estimates for the factor loadings for each method.

##### 4.2.4.1 Stack FA and Ind FA

For both Stack FA and Ind FA, post‐processing begins by extracting the posterior samples of the loading matrices. To obtain a representative point estimate of the loading matrix Φ, we apply orthogonal Procrustes (OP) alignment across the posterior samples. The common covariance matrix is then estimated as $\Phi \Phi^{⊤}$, while the marginal covariance matrix is computed as the average across posterior draws.

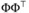

To estimate the number of latent factors, we perform eigenvalue decomposition (EVD) on the marginal covariance matrix and retain the number of components required to explain a pre‐specified proportion of variance. Based on this estimate, the models are refit using the selected number of factors, and the final results are then obtained.

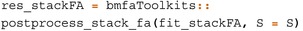

The estimated number of common factors for Stack FA is K=4.

We then re‐fit Stack FA using the initial fit as a starting point, and obtain the final posterior summaries using the following code:

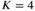

A similar procedure is applied for Ind FA. First, we post‐process the output to estimate the factor loadings, marginal covariance matrices, and noise variances. The number of factors for each study is estimated by applying eigenvalue decomposition to the covariance matrices:

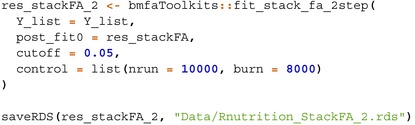

The estimated values of $J_{s}$ for Ind FA are 4, 5, 5, 5, 4, and 4.

The model is then re‐fit for each study using the corresponding number of factors:

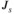

##### 4.2.4.2 PFA

The following code performs post‐processing for the PFA model.

We first estimate the number of factors K by calculating the number of columns in the loading matrix from each MCMC sample. We identify the mode of these values and retain only those posterior samples corresponding to this modal K for downstream analysis.

To obtain the common loading matrix, we multiply the loading matrix Φ by the square root of the latent variance matrix $V^{1/2}$ for each retained sample. Although the average of these transformed matrices $\Phi V^{1/2}$can be used as a point estimate, we apply orthogonal Procrustes (OP) rotation to account for identifiability issues. For the common covariance, we compute $\Phi V\Phi^{⊤}$ at each iteration, and average across samples. A similar procedure is applied for the study‐specific and marginal covariance matrices.

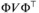

##### 4.2.4.3 MOM‐SS

For MOM‐SS, the common factor loading matrix Φ is directly obtained from the fitted output as a post‐processed estimate. The common covariance is computed as $\Phi \Phi^{⊤}$. The marginal covariance matrix $\sum_{s}$ is then calculated by adding the estimated study‐specific residual covariance matrices to the common covariance component. Additionally, the study‐specific intercepts α and the regression coefficients for the known covariates B are extracted from the fitted object.

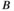

##### 4.2.4.4 SUFA

For SUFA, the common and study‐specific factor loading matrices, along with the common and marginal covariance matrices, are obtained using the lam.est.all(), SUFA_shared_covmat() and sufa_marginal_covs() functions provided by SUFA. The residual covariance is computed by averaging the residuals extracted from the function's output. Study‐specific covariance matrices are calculated by subtracting the common covariance from the marginal covariance. It is important to note that, under the SUFA model formulation, the common covariance matrix is defined as $\Phi \Phi^{⊤}+\sum$.

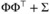

##### 4.2.4.5 BMSFA and CAVI

For BMSFA, the post‐processing procedure follows the same steps as described for Stack FA and Ind FA. We estimate the number of factors by applying eigenvalue decomposition to the common and study‐specific covariance matrices.

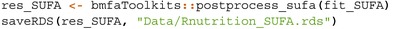

We then re‐run the model using these values: The estimated number of common factors is K=4, and the study‐specific numbers of factors are $J_{s}=2,s=1,\cdots \;,6$. We then re‐run the model using these values:

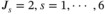

For CAVI, post‐processing is more straightforward; the fitted output provides the loading matrices directly via mean_phi and mean_lambda_s. We then obtain the implied covariances by taking cross‐products of these loading matrices. InbmfaToolkits these steps are implemented in a single function. In addition, CAVI requires refitting the model after estimating the number of factors.

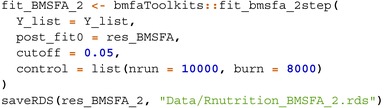

##### 4.2.4.6 BLAST

BLAST directly outputs the estimated loadings in its fitted object via Lambda_mean and Gamma_mean, and the covariance matrices can be obtained by cross‐producting these loading matrices. Moreover, no additional factor‐number estimation is required, as the algorithm selects the dimensions automatically.

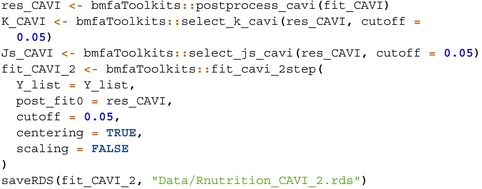

##### 4.2.4.7 Tetris

For Tetris, the combinatorial loadings $\Phi^{*}$ are obtained using the getLambda() function. The common factor loadings Φ are computed as $\Phi^{*}P$, and the study‐specific factor loadings $\Lambda_{s}$ as $\Phi^{*}R_{s}$, where $P+R_{s}=T_{s}$, following the definition in Table 1. The common covariance matrix is computed as $\Phi \Phi^{⊤}$ and the study‐specific covariance matrices as $\Lambda_{s}\Lambda_{s}^{⊤}$, and the marginal covariance matrices as $\Phi T_{s}\Phi^{⊤}+\Psi_{s}$, for each s=1,…,S.

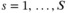

##### 4.2.4.8 Convenient Wrappers in bmfaToolkits

In bmfaToolkits, we provide one‐step functions fit_stack_fa_2step, fit_ind_fa_2step, fit_bmsfa_2step, and fit_cavi_2step for Stack FA, Ind FA, BMSFA, and CAVI that perform the 2‐step refitting workflows automatically. For example,

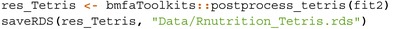

This single function automatically fits an initial (often over‐specified) model, post‐processes the fit to obtain covariance estimates, selects the number of factors, and then refits the model. he returned object contains the final results.

In addition, we provide convenient wrapper functions fit_integrative_fa and postprocess_ integrative_fa that can be used for all methods, with the desired method specified by the user.

#### 4.2.5 Visualization

We visualize the estimated factor loadings using heatmaps, where each row represents a variable (i.e., nutrient) and each column corresponds to a latent factor. To ensure consistency across methods, columns in each loading matrix are ordered according to the proportion of variance explained by each factor, computed as the corresponding eigenvalue divided by the sum of the eigenvalues.

**FIGURE 4:**
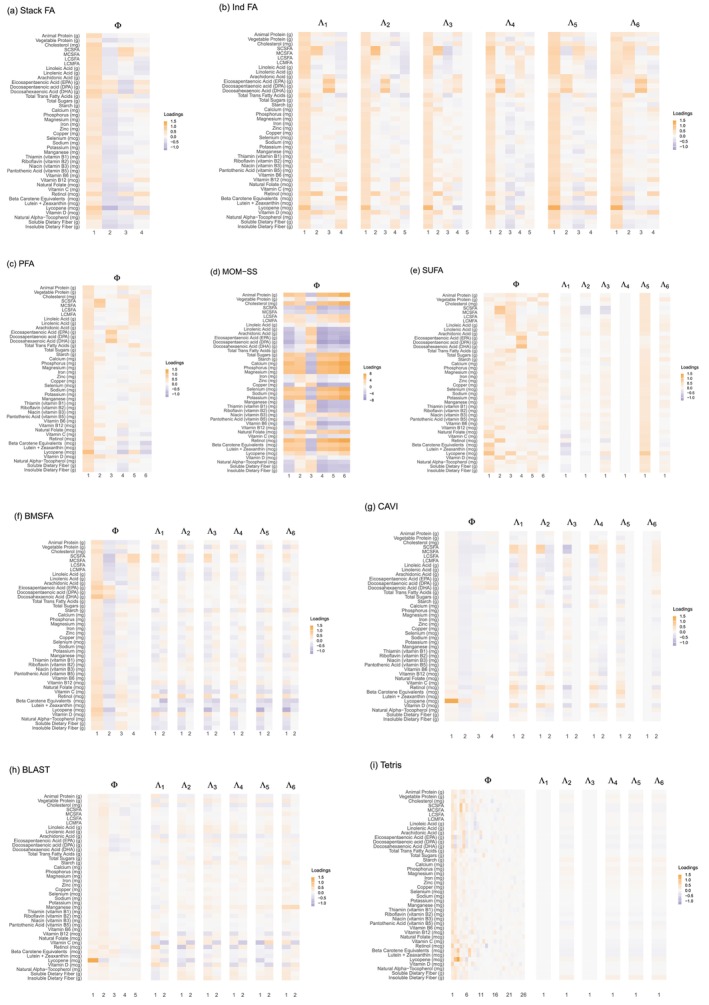
Heatmaps of the factor loading matrices for each method. Rows correspond to nutrients, and columns to latent factors. Loadings in blue (orange) represent negative (positive) associations.

The estimated factor loadings across methods reveal consistent dietary patterns (Figure 4). Factors with high positive loadings on animal protein, saturated fat, and cholesterol likely reflect meat‐heavy dietary habits, whereas those with strong positive loadings on fiber, folate, and plant‐based proteins suggest a plant‐based diet. Similarly, factors dominated by omega‐3 fatty acids and nutrients associated with seafood consumption indicate seafood‐based dietary patterns. Lycopene, a nutrient found in tomatoes, consistently contributes to one of the factors across methods, suggesting a dietary pattern characterized by high tomato intake.

The methods differ notably in the level of sparsity and thus interpretability. Stack FA, Ind FA, and PFA produce clear loading structures, where nutrient groupings are well defined and associated with distinct dietary profiles. In contrast, MOM‐SS estimates more diffuse loadings, with multiple nutrients contributing to each factor, making interpretation more complex. This diffuse structure aligns with the behavior of MOM‐SS's non‐local spike‐and‐slab priors, which encourage a sharp separation between near‐zero and non‐zero loadings. SUFA and BMSFA impose greater sparsity, resulting in more interpretable loadings in which each factor is primarily driven by a small number of nutrients. CAVI and BLAST exhibit a similar pattern to BMSFA, but both allow some loadings to become excessively large. For example, in the first common factor for CAVI and BLAST, the loading associated with lycopene is estimated to be 3.6, where the others fall between 0 and 1. Tetris, on the other hand, tends to overestimate the number of factors, producing nearly empty loaded columns.

The Tetris method required extensive computational time, taking over four days to complete. This is due not only to the large sample size (N) but also to its iterative search for additional common factors. Similar behavior was observed in Simulation Scenario 1, where Tetris overestimated the number of factors under a data‐generating process consistent with a PFA model. To improve computational efficiency in such settings, one may specify a fixed number of factors (i.e., a fixed 𝒯) or adjust the inclusion parameters ($\alpha_{𝒯}$ and $\beta_{𝒯}$). In scenarios such as the nutrition data or Simulation Scenario 1, where the number of observations (N) exceeds the number of variables (P), smaller values of $\alpha_{𝒯}$ and $\beta_{𝒯}$, are recommended to prevent an excessive number of factors.

#### 4.2.6 Model Prediction Accuracy

To evaluate the predictive performance of each model, we compute the mean squared error (MSE) by reconstructing observed data from estimated factor scores and loadings. Each model is trained on a randomly selected 70% of the data, and prediction accuracy is evaluated on the remaining 30%. Using Stack FA in this illustrative example, predictions are obtained as:

(20) $$\begin{array}{cl} {\hat{f}}_{is,(new)} & ={\left({\hat{\Phi }}^{⊤}{\hat{\Psi }}^{-1}\hat{\Phi }\right)}^{-1}{\hat{\Phi }}^{⊤}{\hat{\Psi }}^{-1}y_{is,(new)},\\ {\hat{y}}_{is,(new)} & =\hat{\Phi }{\hat{f}}_{is,(new)}, \end{array}$$

where $\hat{\Phi }$ denotes the factor loadings matrix estimated from the training data, and ${\hat{f}}_{is,(new)}$ is the corresponding factor score estimated using an adaptation of the Bartlett method [76]. MSE calculations for the other methods follow a similar procedure; the formulas for each method are provided in the Supporting Information.

The mean squared error of prediction is then calculated as:

(21) $$\frac{1}{P\sum_{s}^{S}N_{s}}\overset{S}{\underset{s}{\sum }}\overset{N_{s}}{\underset{i}{\sum }}\overset{P}{\underset{p}{\sum }}{\left({\hat{y}}_{isp,(new)}-y_{isp,(new)}\right)}^{2},$$

using the 30% of samples in each study that were held out for testing.

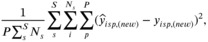

Using the same example, the MSE for Stack FA is computed as follows:

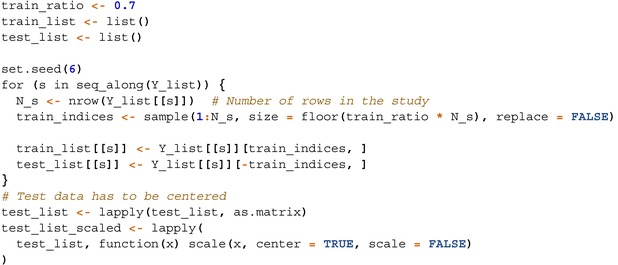

Table 3 reports the MSE for each method.

**TABLE 3:** Estimated numbers of factors and Mean Squared Error (MSE) of different models on the nutrition data.

Model	Estimated K	Estimated $J_{s}$	MSE
Stack FA	4	—	0.514
Ind FA	—	(4, 5, 5, 5, 4, 4)	0.503
PFA	6	—	0.706
MOM‐SS	6	—	0.490
SUFA	6	(1, 1, 1, 1, 1, 1)	0.462
BMSFA	4	(2, 2, 2, 2, 2, 2)	0.473
CAVI	4	(2, 2, 2, 2, 2, 2)	0.165
BLAST	5	(2, 2, 2, 2, 2,2)	0.136
Tetris	26	(0,1,0, 1, 1, 1)	0.318

*Note:* Tetris did not complete within 5 days. K: Numbers of common factors; $J_{s}$: Numbers of study‐specific factors.

The MSE values provide insight into how well each model performs in prediction. CAVI and BLAST outperform the other methods, with MSE values of 0.165 and 0.136, respectively, while estimating fewer factors than others. Tetris achieves the third lowest MSE (0.314), due to its estimation of a large number of factors (K=26). However, as observed in the loading matrices, many of these factors present very small or absent loadings, suggesting that Tetris overfits the data. Following Tetris, SUFA (0.462) and BMSFA (0.473) yield the lowest prediction errors among the remaining models, with a balance between low error and model parsimony. These results highlight their effectiveness in capturing both common and study‐specific dietary patterns.

Conversely, PFA shows the highest MSE (0.706), indicating limited predictive accuracy—likely a consequence of its structural constraint requiring all studies to align with a common reference loading, which may fail to accommodate population‐specific dietary variability.

### 4.3 Case 2: Gene Expression Data Analysis

In this demonstration, we use the curatedOvarianData package [70] to illustrate (1) common gene co‐expression network captured by the shared covariance matrix $\sum_{\Phi }$, and (2) model performance through prediction error, measured by mean squared error (MSE). This dataset includes gene expression microarray data and clinical outcomes for 2970 ovarian cancer patients across 23 studies. The studies vary in terms of sequencing platform, sample size, tumor stage and subtype, survival, and censoring information.

#### 4.3.1 Loading the Data

We begin by loading the curatedOvarianData package, which contains standardized and preprocessed expression data from multiple ovarian cancer studies.

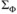

A list of all available datasets can be retrieved using:

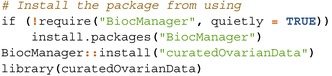

For our analysis, we select four representative studies:

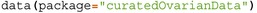

These four datasets have a similar sample size. Across all four studies, the majority of patients are in advanced cancer stages, and the predominant histological subtype is serous carcinoma. However, they differ in sequencing platforms: Operon V3 two‐color, Affymetrix HG‐U133A, Affymetrix HG‐U133 Plus 2.0, and Affymetrix HG‐U133A, respectively.

#### 4.3.2 Pre‐Processing

First, we identify the genes common to the four studies. The featureNames function is used to extract gene names, and exprs retrieves the expression matrices [77].

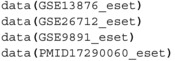

Next, we filter the genes with high variance by using the coefficient of variation (CV). Genes with a CV above a fixed threshold (0.16) in at least one study are retained:

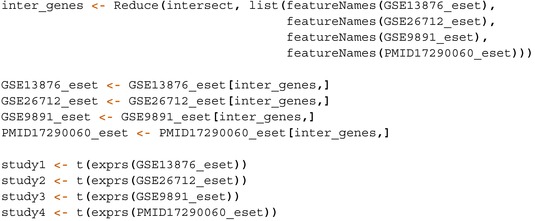

The expression data are then log‐transformed to improve normality and saved in lists of $N_{s}\times P$ matrices:

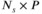

The resulting dataset includes $N_{s}=(157,195,285,117)$ for s=1,2,3,4, with P=1060 genes. We standardize (both center and scale) the data to focus on correlations across genes.

#### 4.3.3 Model Fitting

We proceed to fit the models. Below is an example using Stack FA. For all other methods, we follow the same fitting approach used in the nutrition data analysis (see Section 4.2.3) or refer to the full code available at: https://mavis‐liang.github.io/Bayesian_integrative_FA_tutorial.

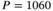

The number of factors is set to $J_{s}=20$ for Ind FA, K=20 for MOM‐SS, PFA, and SUFA, K=20, $J_{s}=4$ for BMSFA and CAVI, and $k_{max}=25$ for BLAST.

#### 4.3.4 Post‐Processing

Post‐processing for obtaining point estimates of the loading and covariance matrices follows the same procedure as in the nutrition case. For Stack FA, Ind FA, BMSFA, and CAVI, this includes an additional step of estimating the number of factors via eigenvalue decomposition (EVD), followed by re‐fitting the model with the estimated number.

For example, with Stack FA:

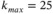

To estimate the number of factors:

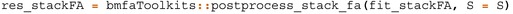

We then re‐run the model with K_StackFA=2:

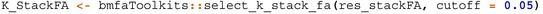

The estimated number of factors for each model is summarized in Table 4.

**TABLE 4:** Estimated numbers of factors and MSE for each model on the gene expression data.

Model	Estimated K	Estimated $J_{s}$	MSE
Stack FA	2	—	0.0208
Ind FA	—	(4, 7, 6, 6)	0.0143
PFA	20	—	0.0161
MOM‐SS	20	—	0.0470
SUFA	19	(4, 4, 4, 4)	0.0131
BMSFA	6	(4, 4, 4, 4)	0.0131
CAVI	6	(4, 4, 3, 3)	0.0139
BLAST	1	(10, 12, 18, 6)	0.0127
Tetris^a^	—	—	—

*Note:* K: Numbers of common factors; $J_{s}$: Numbers of study‐specific factors.

^a^ Tetris did not complete within 5 days.

#### 4.3.5 Visualization

Next, we use Gephi [78] to visualize the common gene co‐expression networks using the estimated common covariances. Edges between two genes are included if the absolute value of their corresponding entry in ${\hat{\sum }}_{\Phi }$ exceeds a threshold. As the estimated covariances differ in magnitude across methods, we apply method‐specific thresholds so that we only keep the top 100–200 prominent correlations for visualization. For instance, the thresholds are 0.55 for PFA, 0.85 for Stack FA, 0.95 for MOM‐SS, 0.28 for SUFA, 0.5 for BMSFA, 0.5 for CAVI, and 0.25 for BLAST.

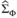

**FIGURE 5:**
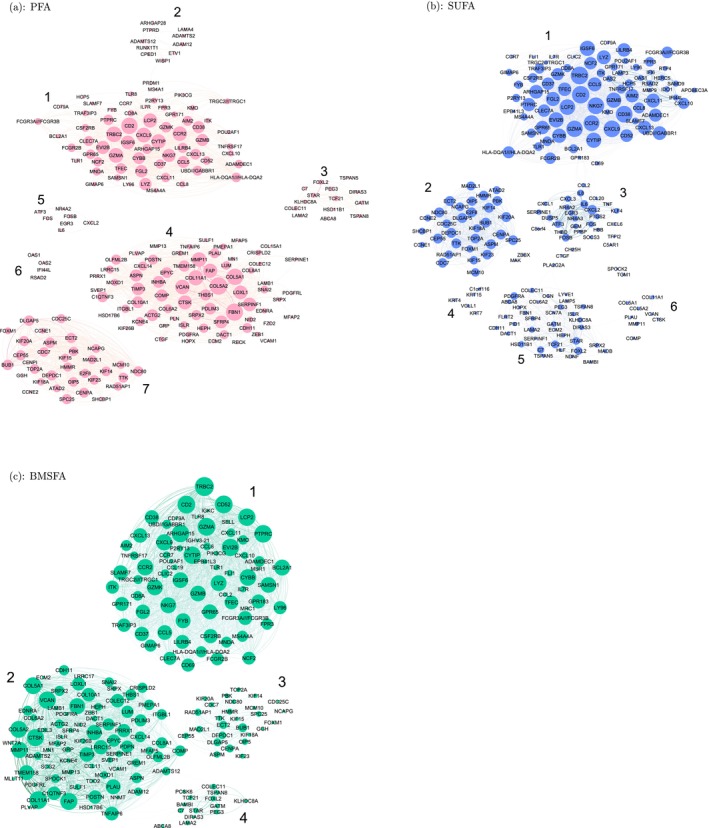
Shared gene co‐expression network based on ${\hat{\sum }}_{\Phi }$ across the ovarian cancer studies for different methods obtained using Gephi [78]. We include edges between two genes if the corresponding element in the shared covariance matrix ${\hat{\sum }}_{\Phi }$ is greater than a threshold in absolute value. Numbers refer to clusters identified by the Yifan Hu [79] algorithm. Results of other methods can be found in the Supporting Information.

Figure 5 displays the shared gene co‐expression network derived from the PFA, SUFA, and BMSFA models. Ind FA is excluded as it does not estimate common covariance. CAVI and BLAST are also omitted from the figure. Stack FA and MOM‐SS produce overly dense networks: Over 200 genes form a single large cluster due to uniformly high covariances (>0.9), while CAVI and BLAST estimate the associations uniformly low. For BLAST, this may be because it identifies only one common factor (see Table 4). For these four methods, the resulting networks contain only a single large gene cluster; therefore, we report the corresponding plots in the Supporting Information.

Tetris is excluded because the model did not complete within six days. Despite the long runtimes for PFA (4 days) and SUFA (over 10 h), we include their results for comparative purposes.

In the co‐expression network (Figure 5), nodes represent genes, and edges correspond to the gene‐gene covariances. Node size reflects the number of connections, and edge darkness reflects the magnitude of covariance. Genes grouped in the same cluster may be co‐regulated, share functional pathways, or reflect common transcriptional programs. These clusters highlight biological processes relevant to ovarian cancer progression and may help identify candidate prognostic markers [80].

Cluster structure differs slightly across methods. BMSFA tends to group more genes into a single large cluster, while PFA and SUFA yield several clusters. These differences can be related to the distinct definitions of $\sum_{\Phi }$: In PFA, $\sum_{\Phi }$ is the marginal covariance of the first study (GSE13876_eset); in SUFA, it is defined as $\Phi \Phi^{⊤}+\Psi$; and in BMSFA, it corresponds to $\Phi \Phi^{⊤}$. The number of extracted factors and estimation techniques further contribute to these structural differences. Nonetheless, several recurrent clusters across methods point to a robust biological signal.

*Immune‐related*: Cluster 1 in PFA, SUFA, and BMSFA includes key immune‐regulatory genes such as *CXCL9*, *CXCL10*, *CXCL11*, *CXCL13*, and *CCR2*. These chemokines and their receptors are critical in immune cell recruitment and activation, mediating antitumor immunity [81, 82, 83].

*Extracellular matrix (ECM) organization related*: Cluster 4 (PFA), Cluster 6 (SUFA), and Cluster 2 (BMSFA) contain genes involved in extracellular matrix (ECM) organization and remodeling, including *COL10A1*, *COL11A1*, *COL5A1*, *POSTN*, *VCAN*, *TIMP3*, *THBS1*, *FAP*, and *LOXL1*. These genes regulate ECM stiffness and integrity—factors known to influence tumor progression and metastasis [84, 85].

#### 4.3.6 Model Prediction Accuracy

As in the nutrition data analysis, model prediction accuracy is evaluated using mean squared error (MSE). Each dataset is randomly partitioned into a 70% training set and a 30% test set. Models are fit on the training data using the estimated number of factors reported in Table 4. Factor scores for the test set are then estimated using the fitted loadings and residual covariances and used to reconstruct the observed test data. Tetris is excluded from this comparison, for high intensive computational time.

As shown in Table 4, all models demonstrate a good fit to the gene expression data. BLAST achieves the lowest MSE (0.0127), following by SUFA (0.0131), BMSFA (0.0131) and CAVI (0.0139).

Although the MSE values are close, BMSFA and CAVI are preferable due to their more parsimonious factor structure—requiring fewer common factors than BLAST and SUFA.

PFA also yields a competitive MSE but relies on a large number of factors (K=20), suggesting that the default cutoff parameter (0.001) may be too permissive for this dataset. Stack FA and MOM‐SS show higher MSE values. For Stack FA, this may reflect a limited ability to capture the complex covariance structure of the gene expression data. For MOM‐SS, convergence of the EM algorithm can be more challenging in this high‐dimensional setting.

## 5 Discussion

This tutorial reviewed a range of Bayesian integrative factor models for analyzing multi‐study, high‐dimensional data, motivated by the growing need to jointly analyze complex datasets arising across studies and platforms. Such settings are increasingly common in genomics, nutrition, and epidemiology, where principled approaches are needed to disentangle shared and study‐specific structure while remaining computationally feasible and interpretable.

Our comparative evaluations highlight clear trade‐offs among modeling flexibility, computational scalability, and inferential robustness. No single method dominates across all scenarios, underscoring the importance of aligning model choice with the scientific goals, study design, and data characteristics. For example, Stack FA and Ind FA provide fast baselines, but their limited ability to represent both shared and study‐specific variation can reduce robustness when study‐level sample sizes are small and may yield biased covariance estimates when heterogeneity is substantial. In contrast, more expressive models improve accuracy and interpretability, but typically require greater computation and more extensive post‐processing.

From a scalability perspective, SUFA offers a favorable balance of accuracy and efficiency in small‐to‐moderate dimensions, aided by its efficient HMC sampler. CAVI‐based approaches can also be computationally attractive in high‐dimensional settings; however, they may introduce approximation bias, underestimate posterior uncertainty, and exhibit sensitivity to initialization. MOM‐SS enables rapid estimation via EM‐based inference, making it appealing in large‐scale settings, although it may overestimate factor complexity in more challenging scenarios. BMSFA and PFA aim to improve inferential robustness by relaxing identifiability constraints or reducing rotational ambiguity, respectively, but both can become computationally demanding as the number of variables increases. Tetris is the most flexible approach, automatically capturing fully shared, partially shared, and study‐specific factors; however, its nonparametric nature makes it prohibitively expensive in large‐scale applications without substantial computational resources.

Identifiability and post‐processing are central practical considerations across methods. Models that impose strong structural constraints, such as SUFA, can gain computational efficiency but may sacrifice precision in estimating study‐specific effects. Approaches such as PFA can simplify post‐processing by anchoring studies to a reference structure, though indirect estimation of study‐specific effects and the need to estimate full perturbation matrices may limit scalability. BMSFA mitigates identifiability issues using two alternative post‐processing procedures: (1) a singular value decomposition (SVD) of the posterior‐mean covariance matrix obtained by averaging Gibbs sampler draws, and (2) an orthogonal Procrustes (OP) approach [63], which recovers the loading matrices ex post by post‐processing MCMC output from an unrestricted model fit. Both options can increase implementation complexity and may complicate interpretation.

Selecting the number of latent factors remains a key challenge. With the exception of Tetris, most methods require either pre‐specification or iterative selection strategies. Eigenvalue‐based screening followed by refitting is common but can be unstable, while adaptive procedures embedded in models such as MOM‐SS and SUFA may lack precision. Practitioners therefore face a practical balance between model complexity and interpretability: Rich factor structures risk overfitting and instability, whereas overly sparse representations may obscure meaningful latent structure.

Taken together, these considerations suggest a practical workflow for Bayesian integrative factor analysis: (i) careful data preprocessing and harmonization across studies; (ii) model choice guided by sample size, dimensionality, and computational constraints; (iii) estimation with appropriate tuning and post‐processing to address factor alignment and identifiability; and (iv) interpretation via covariance networks, latent factor summaries, or predictive modeling, optionally supported by predictive performance metrics such as mean squared error.

Overall, Bayesian integrative factor models provide a principled framework for uncovering latent structure in multi‐study settings. By explicitly weighing estimation accuracy, interpretability, and computational feasibility, practitioners can select methods that are well matched to their data and scientific objectives. This tutorial is intended to serve both as a practical guide for applied researchers and as a foundation for future methodological developments in integrative latent variable modeling.

## Funding

This work was supported by the Ministero dell'Istruzione, dell'Università e della Ricerca (Grant Programma per Giovani Ricercatori Rita Levi‐Montalcini).

## Conflicts of Interest

The authors declare no conflicts of interest.

## Supporting information




**Data S1.** Supporting Information.

## Data Availability

The data that support the findings of this study are available in curatedOvarianData at: https://bioconductor.org/packages/release/data/experiment/html/curatedOvarianData.html. These data were derived from the following resources available in the public domain: curatedOvarianData, https://bioconductor.org/packages/release/data/experiment/html/curatedOvarianData.html.
