# Supplementary material for: A Tutorial on Bayesian Multi‐Study Factor Analysis With Applications in Nutrition and Genomics
Source: Stat Med. 2026 Apr 24;45:e70531. doi: 10.1002/sim.70531 (PMC13109005; doi:10.1002/sim.70531)
Supplement: Supplementary file 1 — Data S1. Supporting Information. [file SIM-45-0-s001.pdf]

Supplement to:

A Tutorial on Bayesian Multi-Study Factor Analysis with Applications in Nutrition and Genomics.

Mavis Liang, Blake Hansen, Alejandra Avalos-Pacheco, Roberta De Vito*
*Correspondence: roberta.devito@uniroma1.it

April 1, 2026

Contents

Appendix 1: Glossary **2**

Appendix 2: Summary of the Input, Output and Post-processing for Different Methods **2**

Appendix 3: More results in simulation **4**

Appendix 4: Calculating MSE **6**

Appendix 5: More results in gene application **7**

Appendix 1: Glossary

Term or Abbreviation	Definition
FA	Standard factor analysis. It models the multivariate $\mathbf{y}_{is}$ with a linear combination of latent factors and random errors.
loadings	Entries in the loadings matrix of a factor model quantifying the relationship between high-dimensional variables to the low-dimensional latent variables.
batch-effect	Unwanted technical effect when the experiments are taken with different
idiosyncratic errors	Variances of the error terms of different factors are defined differently
Prior	A pre-defined distribution that we believe the estimator is following.
identifiability	In the context of factor analysis, identifiability issue refers to the non-uniqueness of loadings and factor scores that can satisfy the model likelihoods.
rotational ambiguity	Multiplying the loadings matrix and factor matrix with a orthogonal matrix results in the rotations of the loadings and factor matrix that can still satisfy model likelihoods.
Varimax	Rotation to the loadings matrix which ensure that variables are only contributing to only a few factors, to enhance interpretability for loadings
OP	Orthogonal Procrustes. Another rotational techniques
Stack FA	Stacking the multi-study data into a single study and applying standard FA.
Ind FA	Applying standard FA to each multi-study datasets separately.
PFA	Perturbed factor analysis.
MOM-SS	The Bayesian regression factor model with a moment-based non-local spike-and-slab normal prior.
SUFA	Subspace factor analysis.
MSFA	Multi-study factor analysis.
BMSFA	Bayesian multi-study factor analysis.
EVD	Eigen value decomposition.
MCMC	Monte-carlo.
EM	Expection-maximization algorithm.
HMC	Hamilton-Monte-Carlo.
MGPS	Multiplicative Gamma shrinkage prior.

Appendix 2: Summary of the Input, Output and Post-processing for Different Methods

Table 1: *Summary of the required input, fitted output, and post-processings for desired estimands.*

Model	Input	Fitted output	Post-processing
**Stack FA**	$\mathbf{Y}$ of vertically stacked<br>$\mathbf{Y}_1, \cdots, \mathbf{Y}_S$;<br>No. of common factors K;	Posterior samples of $\Phi : \Phi^{(1)}, \Phi^{(2)}, \ldots, \Phi^{(M)}$.<br>Posterior samples of $\Psi : \Psi^{(1)}, \Psi^{(2)}, \ldots, \Psi^{(M)}$.	$\widehat{\Phi}$:OP the posterior samples of Φ.<br>$\widehat{\Sigma_\Phi}$:$\widehat{\Phi}\widehat{\Phi}^\top$.<br>$\widehat{\Sigma_s}$:Obtain $\widehat{\Psi}$ for each study by averaging the posterior samples of $\Psi^{(i)}, i = 1, \cdots, M$.<br>Then $\widehat{\Sigma_s} = \widehat{\Phi}\widehat{\Phi}^\top + \widehat{\Psi}$.
**Ind FA**	Lists of $\mathbf{Y}_1, \cdots, \mathbf{Y}_S$;<br>$J_1, \cdots, J_S$;	Posterior samples of $\Lambda_s : \Lambda_s^{(1)}, \Lambda_s^{(2)}, \ldots, \Lambda_s^{(M)}$.<br>Posterior samples of $\Psi_s : \Psi_s^{(1)}, \Psi_s^{(2)}, \ldots, \Psi_s^{(M)}$.	$\widehat{\Lambda_s}$:OP the posterior samples of Λ_s.<br>$\widehat{\Sigma_{\Lambda_s}}$:$\widehat{\Lambda_s}\widehat{\Lambda_s}^\top$.<br>$\widehat{\Sigma_s}$:Obtain $\widehat{\Psi_s}$ for each study by averaging the posterior samples of $\Psi_s^{(i)}, i = 1, \cdots, M$.<br>Then $\widehat{\Sigma_s} = \widehat{\Lambda_s}\widehat{\Lambda_s}^\top + \widehat{\Psi_s}$.
**PFA**	Lists of $\mathbf{Y}_1, \cdots, \mathbf{Y}_S$;<br>$K$;<br>$\alpha_Q$ (Default=0.01);	Posterior samples of $\hat{K} : \hat{K}^{(1)}, \hat{K}^{(2)}, \ldots, \hat{K}^{(M)}$.<br>Posterior samples of $\Phi : \Phi^{(1)}, \Phi^{(2)}, \ldots, \Phi^{(M)}$.<br>Posterior samples of $E' : E'^{(1)}, E'^{(2)}, \ldots, E'^{(M)}$, where elements in E' are the square root of the elements in E in Table 1.<br>Posterior samples of $Q_s : Q_s^{(1)}, Q_s^{(2)}, \ldots, Q_s^{(M)}$.<br>Posterior samples of $\Psi' : \Psi'^{(1)}, \Psi'^{(2)}, \ldots, \Psi'^{(M)}$, where elements in Ψ' are the square root of the elements in Ψ in Table 1.	$\hat{K}$: Use the mode of $\hat{K}^{(1)}, \hat{K}^{(2)}, \ldots, \hat{K}^{(M)}$<br>$\widehat{\Phi} : \Phi^{(i)}E'^{(i)}$ for $i = 1, 2, \ldots, M,$ then OP the sequence.<br>$\widehat{\Sigma_\Phi} : \Phi^{(i)}E'^{(i)^2}\Phi^{(i)^\top} + \Psi'^{(i)^2}$ for $i = 1, 2, \ldots, M,$ then average.<br>$\widehat{\Sigma_{\Lambda_s}}$: For $i = 1, 2, \ldots, M,$ let $\Sigma_\Phi^{(i)} = \Phi^{(i)}E'^{(i)^2}\Phi^{(i)^\top} + \Psi'^{(i)^2}$, and $\Sigma_{\Lambda_s}^{(i)} = Q_s^{(i)^{-1}}\Sigma_\Phi^{(i)}(Q_s^{(i)^{-1}})^\top - \Sigma_\Phi^{(i)}$.<br>Then $\widehat{\Sigma_{\Lambda_s}}$ is the average of $\Sigma_{\Lambda_s}^{(i)}$.<br>$\widehat{\Sigma_s}$: For $i = 1, 2, \ldots, M,$ let $\Sigma_s^{(i)} = \Phi^{(i)}E'^{(i)^2}\Phi^{(i)^\top} + \Psi'^{(i)^2}$, and $\Sigma_s^{(i)} = \Sigma_s^{(i)} + Q_s^{(i)^{-1}}\Sigma_\Phi^{(i)}(Q_s^{(i)^{-1}})^\top$.<br>Then $\widehat{\Sigma_s}$ is the average of $\Sigma_s^{(i)}$.
**MOM-SS**	$\mathbf{Y}$ of vertically stacked<br>$\mathbf{Y}_1, \cdots, \mathbf{Y}_S$;<br>$X$;<br>$M$;<br>$K$;	Point estimates of Φ.<br>Poit estimates of Ψ_s	$\widehat{\Phi}$: Point estimate of Φ.<br>$\widehat{\Sigma_\Phi} : \widehat{\Phi}\widehat{\Phi}^\top$.<br>$\widehat{\Sigma_s} : \widehat{\Phi}\widehat{\Phi}^\top + \widehat{\Psi_s}$.
**SUFA**	Lists of $\mathbf{Y}_1, \cdots, \mathbf{Y}_S$;<br>$K$;	Posterior samples of $\Phi : \Phi^{(1)}, \Phi^{(2)}, \ldots, \Phi^{(M)}$.<br>Posterior samples of $A_s : A_s^{(1)}, A_s^{(2)}, \ldots, A_s^{(M)}$.<br>Posterior samples of $\Psi : \Psi^{(1)}, \Psi^{(2)}, \ldots, \Psi^{(M)}$.	$\widehat{\Phi}$: Use command `lam.est()` in SUFA package.<br>$\widehat{\Sigma_\Phi}$: Use command `SUFA_shared_covmat()` in SUFA package. It computes the posterior means of $\Phi\Phi^\top + \Psi$.<br>$\widehat{\Sigma_{\Lambda_s}}$: Use command `sufa_marginal_covs()` and `SUFA_shared_covmat()` in SUFA package. Then subtract the former outcomes with the latter.<br>$\widehat{\Lambda_s}$: Use command `lam.est.all()` in SUFA package.<br>$\widehat{\Sigma_s}$: Use command `sufa_marginal_covs()` in SUFA
**BMSFA**	Lists of $\mathbf{Y}_1, \cdots, \mathbf{Y}_S$;<br>$K$;<br>$J_1, \cdots, J_S$;	Posterior samples of $\Phi : \Phi^{(1)}, \Phi^{(2)}, \ldots, \Phi^{(M)}$.<br>Posterior samples of $\Lambda_s : \Lambda_s^{(1)}, \Lambda_s^{(2)}, \ldots, \Lambda_s^{(M)}$.<br>Posterior samples of $\Psi_s : \Psi_s^{(1)}, \Psi_s^{(2)}, \ldots, \Psi_s^{(M)}$.	$\widehat{\Phi}$: OP the posterior samples of Φ.<br>$\widehat{\Sigma_\Phi} : \widehat{\Phi}\widehat{\Phi}^\top$.<br>$\widehat{\Lambda_s}$: OP the posterior samples of Λ_s.<br>$\widehat{\Sigma_{\Lambda_s}} : \widehat{\Lambda_s}\widehat{\Lambda_s}^\top$.<br>$\widehat{\Sigma_s}$: Obtain $\widehat{\Psi_s}$ for each study by averaging the posterior samples of $\Psi_s^{(i)}$,<br>$i = 1, \cdots, M$. Then $\widehat{\Sigma_s} = \widehat{\Phi}\widehat{\Phi}^\top + \widehat{\Lambda_s}\widehat{\Lambda_s}^\top + \widehat{\Psi_s}$.
**CAVI**	Lists of $\mathbf{Y}_1, \cdots, \mathbf{Y}_S$;<br>$K$;<br>$J_1, \cdots, J_S$;	$\widehat{\Phi}$: Point estimate of Φ.<br>$\widehat{\Lambda_s}$: Point estimate of Λ_s.<br>$\widehat{\Psi_s}$: Point estimate of Ψ_s.	$\widehat{\Sigma_\Phi} : \widehat{\Phi}\widehat{\Phi}^\top$.<br>$\widehat{\Sigma_{\Lambda_s}} : \widehat{\Lambda_s}\widehat{\Lambda_s}^\top$.<br>$\widehat{\Sigma_s} : \widehat{\Phi}\widehat{\Phi}^\top + \widehat{\Lambda_s}\widehat{\Lambda_s}^\top + \widehat{\Psi_s}$.
**BLAST**	Lists of $\mathbf{Y}_1, \cdots, \mathbf{Y}_S$;<br>$K$;<br>$J_1, \cdots, J_S$;<br>Or $K_{max} = K + \sum_{s=1}^{S} J_s$ alone.	$\widehat{\Phi}$: Point estimate of Φ.<br>$\widehat{\Lambda_s}$: Point estimate of Λ_s.<br>$\widehat{\Sigma_\Phi}$: Point estimate of Σ_Φ.<br>$\widehat{\Sigma_{\Lambda_s}}$: Point estimate of Σ_{Λ_s}.<br>Sequence of $\Psi : \Psi^{(1)}, \Psi^{(2)}, \ldots, \Psi^{(M)}$.	$\widehat{\Sigma_s}$: Obtain $\widehat{\Psi_s}$ for each study by averaging the posterior samples of $\Psi_s^{(i)}$,<br>$i = 1, \cdots, M$. Then $\widehat{\Sigma_s} = \widehat{\Sigma_\Phi} + \widehat{\Sigma_{\Lambda_s}} + \widehat{\Psi}$.
**Tetris**	Lists of $\mathbf{Y}_1, \cdots, \mathbf{Y}_S$	Point estimate of $\mathcal{T} : \widehat{\mathcal{T}}$.<br>Posterior samples of $\Phi^* : \Phi^{*(1)}, \Phi^{*(2)}, \ldots, \Phi^{*(M)}$.<br>Posterior samples of $\Psi_s : \Psi_s^{(1)}, \Psi_s^{(2)}, \ldots, \Psi_s^{(M)}$.	$\widehat{\Phi}$: First we use command `getLambda()` in Tetris package to get point estimates of Φ^*, denoted $\widehat{\Phi^*}$. Then $\widehat{\Phi} = \widehat{\Phi^*}[, \texttt{colSums}(\widehat{\mathcal{T}}) == S]$.<br>$\widehat{\Sigma_\Phi} : \widehat{\Phi}\widehat{\Phi}^\top$.<br>$\widehat{\Lambda_s}$: Construct $\widehat{T_s}$ and $\widehat{P}$ with $\widehat{\mathcal{T}}$ using the definition in Table 1, and let $\widehat{R_s} = \widehat{T_s} - \widehat{P}$.<br>Then select the non-zero columns of $\{\widehat{\Phi^*}\widehat{R_s}\}$ as $\widehat{\Lambda_s}$.<br>$\widehat{\Sigma_{\Lambda_s}} : \widehat{\Lambda_s}\widehat{\Lambda_s}^\top$.<br>$\widehat{\Sigma_s}$: Construct $\widehat{T_s}$ with $\widehat{\mathcal{T}}$ using the definition in Table 1, and calculate the average of $\Psi_s^{(i)}, i = 1, \cdots, M,$ denoted $\widehat{\Psi_s}$. Then $\widehat{\Sigma_s} = \widehat{\Phi^*}T_s\widehat{\Phi^*}^\top + \widehat{\Psi_s}$.

Appendix 3: More results in simulation

Below we present some supplementary results for the simulations, involving Frobenius Norm which also measures the similarities of the two matrices X and Y, which is defined as the square root of the sum of the absolute squares of the elements in $X - Y$:

$$\mathrm{FN}(X, Y) = \sqrt{\sum_{i}^{n} \sum_{j}^{m} |a_{ij}|^2} \tag{1}$$

where a_{ij} are the elements in matrix $X - Y$. Therefore, FN ranges from 0 to infinity, with the smaller the value, the more similarities between the two matrices.

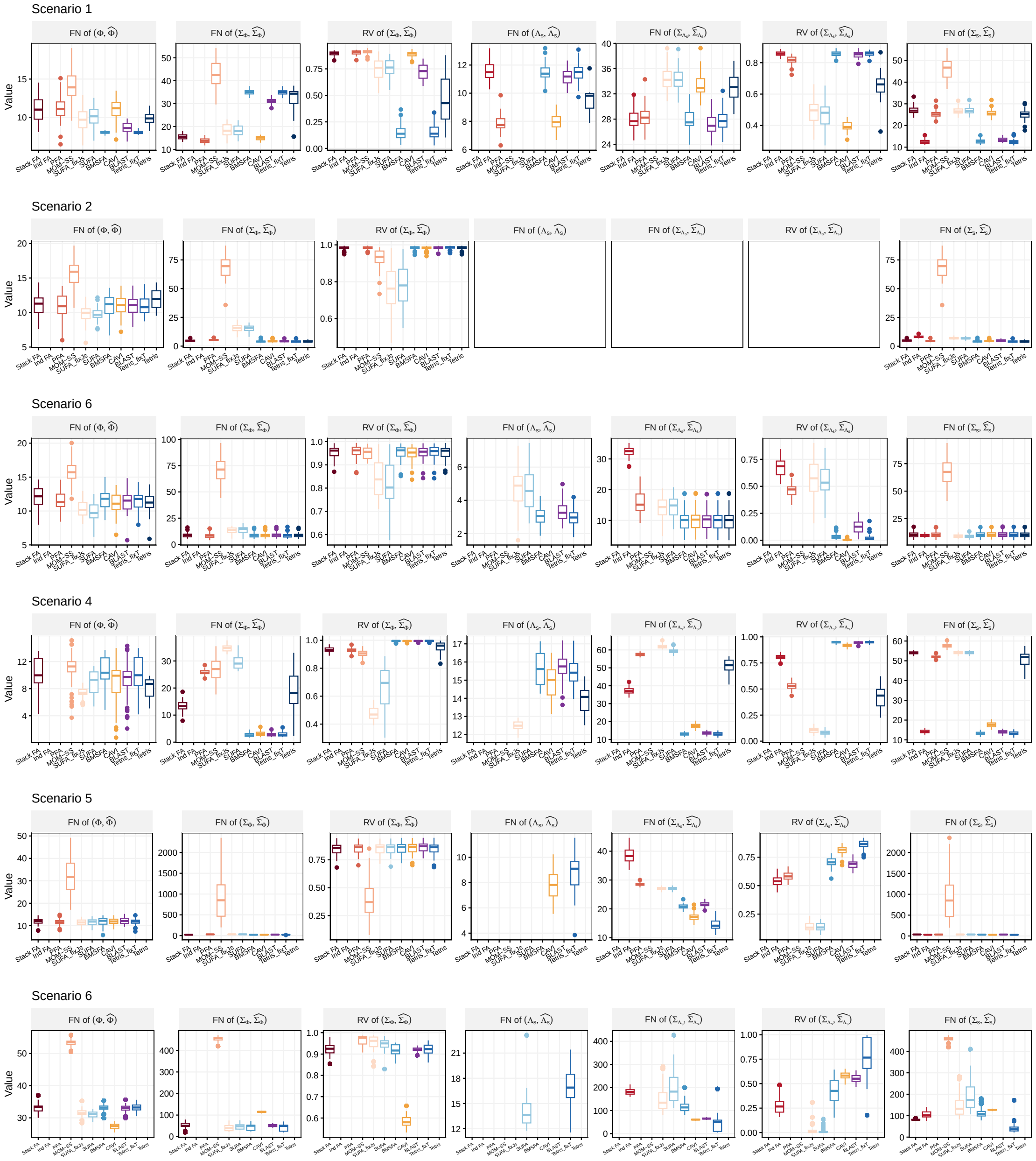

Figure 1: *Measuring accuracy of different Bayesian integrative factor models in estimating factor loadings and marginal covariance matrices using Frobenius Norm (FN) and RV.*

Appendix 4: Calculating MSE

We use the following formula to reconstruct the factor scores and then the test set data:

- Stack FA: $\widehat{\mathbf{f}}_{is,(new)} = (\widehat{\Phi}^\top \widehat{\Psi}^{-1} \widehat{\Phi})^{-1} \widehat{\Phi}^\top \widehat{\Psi}^{-1} \mathbf{y}_{is,(new)}$, and $\widehat{\mathbf{y}}_{is,(new)} = \widehat{\Phi} \widehat{\mathbf{f}}_{is,(new)}$.
- Ind FA: $\widehat{\mathbf{l}}_{is,(new)} = (\widehat{\Lambda_s}^\top \widehat{\Psi_s}^{-1} \widehat{\Lambda_s})^{-1} \widehat{\Lambda_s}^\top \widehat{\Psi_s}^{-1} \mathbf{y}_{is,(new)}$, and $\widehat{\mathbf{y}}_{is,(new)} = \widehat{\Lambda_s} \widehat{\mathbf{l}}_{is,(new)}$.
- PFA: $\widehat{\mathbf{f}}_{is,(new)} = (\widehat{\Phi}^\top \widehat{\Psi}^{-1} \widehat{\Phi})^{-1} \widehat{\Phi}^\top \widehat{\Psi}^{-1} \widehat{Q_s} \mathbf{y}_{is,(new)}$, and $\widehat{\mathbf{y}}_{is,(new)} = \widehat{Q_s}^{-1} \widehat{\Phi} \widehat{\mathbf{f}}_{is,(new)}$.
- MOM-SS: $\widehat{\mathbf{f}}_{is,(new)} = (\widehat{\Phi}^\top \widehat{\Psi_s}^{-1} \widehat{\Phi})^{-1} \widehat{\Phi}^\top \widehat{\Psi_s}^{-1} (\mathbf{y}_{is,(new)} - \widehat{\alpha_\mathbf{s}} - \widehat{\beta} \mathbf{x}_{is,(new)})$, and $\widehat{\mathbf{y}}_{is,(new)} = \widehat{\alpha_\mathbf{s}} + \widehat{\beta} \mathbf{x}_{is,(new)} + \widehat{\Phi} \widehat{\mathbf{f}}_{is,(new)}$.
- SUFA: let $\Omega = [\widehat{\Phi}, \widehat{\Phi} \widehat{A_s}]$, then $\begin{bmatrix} \widehat{\mathbf{f}}_{is,(new)} \\ \widehat{\mathbf{l}}_{is,(new)} \end{bmatrix} = (\Omega^\top \widehat{\Psi}^{-1} \Omega)^{-1} \Omega^\top \widehat{\Psi}^{-1} \mathbf{y}_{is,(new)}$, and $\widehat{\mathbf{y}}_{is,(new)} = \widehat{\Phi} \widehat{\mathbf{f}}_{is,(new)} + \widehat{\Phi} \widehat{A_s} \widehat{\mathbf{l}}_{is,(new)}$.
- BMSFA/CAVI/BLAST: let $\Omega = [\widehat{\Phi}, \widehat{\Lambda_s}]$, then $\begin{bmatrix} \widehat{\mathbf{f}}_{is,(new)} \\ \widehat{\mathbf{l}}_{is,(new)} \end{bmatrix} = (\Omega^\top \widehat{\Psi_s}^{-1} \Omega)^{-1} \Omega^\top \widehat{\Psi_s}^{-1} \mathbf{y}_{is,(new)}$, and $\widehat{\mathbf{y}}_{is,(new)} = \widehat{\Phi} \widehat{\mathbf{f}}_{is,(new)} + \widehat{\Lambda_s} \widehat{\mathbf{l}}_{is,(new)}$.
- Tetris: Let $\Omega = [\widehat{\Phi^* T_s}] = [\widehat{\Phi}, \widehat{\Lambda_s}]$, then $(\Omega^\top \widehat{\Psi_s}^{-1} \Omega)^{-1} \Omega^\top \widehat{\Psi_s}^{-1} \mathbf{y}_{is,(new)}$, and $\widehat{\mathbf{y}}_{is,(new)} = \widehat{\Phi^* T_s} \widehat{\mathbf{f}}_{is,(new)} = \Omega \widehat{\mathbf{f}}_{is,(new)}$.

Appendix 5: More results in gene application

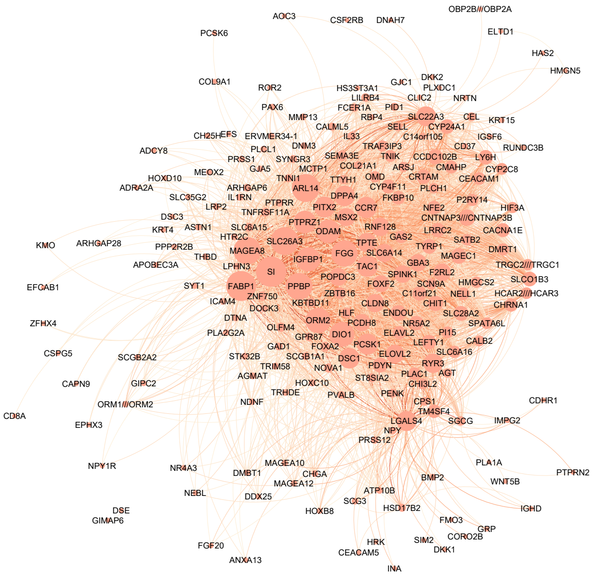

Figure 2: Gene co-expression network ($\Phi\Phi^{\top}$) estimated by Stack FA

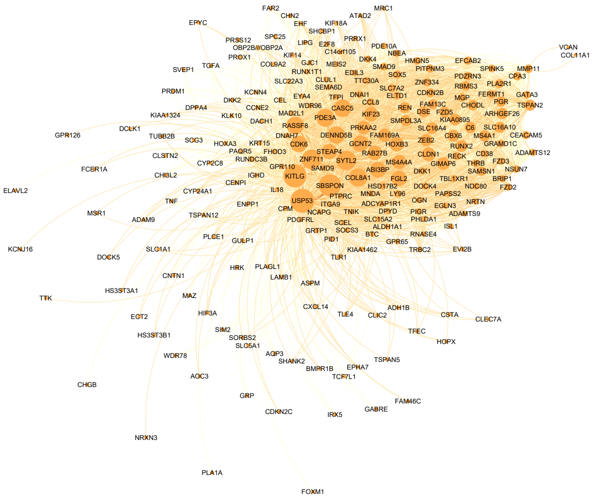

Figure 3: Gene co-expression network ($\Phi\Phi^\top$) estimated by MOM-SS

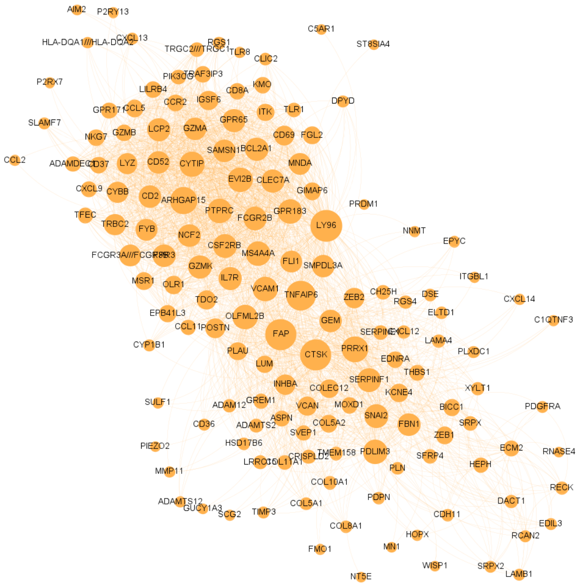

Figure 4: Gene co-expression network ($\Phi\Phi^\top$) estimated by CAVI

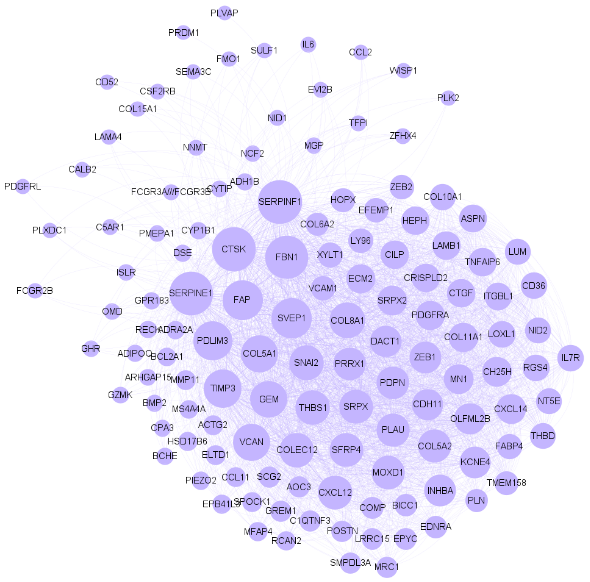

Figure 5: Gene co-expression network ($\Phi\Phi^\top$) estimated by BLAST
